# MAEL in human cancers and implications in prognostication and predicting benefit from immunotherapy over VEGFR/mTOR inhibitors in clear cell renal cell carcinoma: a bioinformatic analysis

**DOI:** 10.18632/aging.205470

**Published:** 2024-01-31

**Authors:** Jin Tao, Jinshan Cui, Yu Xu, Yafeng Fan, Guodong Hong, Qiaoxia Zhou, Guoqiang Wang, Leo Li, Yusheng Han, Chunwei Xu, Wenxian Wang, Shangli Cai, Xuepei Zhang

**Affiliations:** 1 Department of Urology, The First Affiliated Hospital of Zhengzhou University, Zhengzhou, Henan, China; 2 Burning Rock Biotech, Guangzhou, Guangdong, China; 3 Institute of Basic Medicine and Cancer (IBMC), Chinese Academy of Sciences, Hangzhou, Zhejiang, China; 4 Department of Clinical Trial, The Cancer Hospital of the University of Chinese Academy of Sciences (Zhejiang Cancer Hospital), Hangzhou, Zhejiang, China

**Keywords:** MAEL, pan-cancer analysis, clear cell renal cell carcinoma, prognosis, immunotherapy

## Abstract

*Maelstrom* (*MAEL)*, a novel cancer/testis-associated gene, may facilitate the initiation and progression of human malignancies, warranting comprehensive investigations. Single-cell and tissue-bulk transcriptomic data demonstrated higher *MAEL* expression in testis (spermatogonia/spermatocyte), kidney (proximal tubular cell), and brain (neuron/astrocyte), and corresponding cancers, including testicular germ cell tumor, glioma, papillary renal cell carcinoma, and clear cell renal cell carcinoma (ccRCC). Of these cancers, only in ccRCC did *MAEL* expression exhibit associations with both recurrence-free survival and overall survival. High *MAEL* expression was associated with an anti-inflammatory tumor immune microenvironment and VEGFR/mTOR activation in ccRCC tissues and high sensitivities to VEGFR/PI3K-AKT-mTOR inhibitors in ccRCC cell lines. Consistent with these, low rather than high *MAEL* expression indicated remarkable progression-free survival benefits from immune checkpoint inhibitor (ICI)-based immunotherapies over VEGFR/mTOR inhibitors in two large phase III trials (JAVELIN Renal 101 and CheckMate-025). *MAEL* is a biologically and clinically significant determinant with potential for prognostication after nephrectomy and patient selection for VEGFR/mTOR inhibitors and immunotherapy-based treatments.

## INTRODUCTION

*Maelstrom* (*MAEL)*, located in 1q24, is an evolutionarily conserved gene first found in *Drosophila* oocyte [1]. The full-length MAEL protein contains a high-mobility group (HMG) domain for DNA binding as well as a novel MAEL-specific domain with a single-stranded RNA (ssRNA)-specific endonuclease activity [2–4]. *MAEL* expression has been discovered by Northern blot in only the testis of normal human tissues [5], while it has been found to be aberrant in numerous cancer cell lines [5, 6]. Unlike in germ cells, where MAEL is a nuage component involved in posttranscriptional piRNA-mediated transposon silencing [7], MAEL has been identified as a component of stress granule (SG) in tumor cells [8], relating to the cellular response to abnormal physiological or pathological conditions, such as hypoxia, oxidative stress, and chemotherapeutic drugs [9].

As a novel cancer/testis-associated gene, *MAEL* is deemed to participate in stem cell self-renewal that favors tumor proliferation [10]. Emerging evidence has revealed its oncogenic mechanisms in cell lines concerning the cancers in breast [6], esophagus [11], stomach [12], colorectum [13], liver [6, 14], ovary [6, 15], and bladder [16], in terms of inducing epithelial-mesenchymal transition (EMT) [11–15], protecting genetic integrity [6], and recruiting myeloid-derived suppressor cells (MDSCs) that leads to an anti-inflammatory tumor immune microenvironment (TIME) [11]. Given the associations of *MAEL* with EMT/stemness and TIME, we hypothesized that MAEL might define a stemness-like and immune-suppressive phenotype associated with the resistance to immune checkpoint inhibitors (ICIs).

In this study, we first delineated the expression landscape of *MAEL* in human normal tissues and cancers, finding high *MAEL* expression in normal tissues such as testis (spermatogonia/spermatocyte), kidney (proximal tubular cell), and brain (neuron/astrocyte), as well as cancers including testicular germ cell tumor (TGCT), glioma, papillary renal cell carcinoma (pRCC), and clear cell renal cell carcinoma (ccRCC). Of these cancers, only in ccRCC did *MAEL* expression appear to be associated with both recurrence-free survival (RFS) and overall survival (OS). In two large phase III trials, JAVELIN Renal 101 and CheckMate-025, high *MAEL* expression was linked with anti-inflammatory TIME and VEGFR/mTOR activation in ccRCC tissues, high sensitivities to VEGFR/PI3K-AKT-mTOR inhibitors in ccRCC cell lines, and poor progression-free survival (PFS) benefits from ICI-based immunotherapies over VEGFR/mTOR inhibitors.

## MATERIALS AND METHODS

### Study design and clinical cohorts

In total, there are six parts in our study, including (i) *MAEL* expression in normal tissues (tissue bulk RNA-seq, Human Protein Atlas [HPA]) and cells (single cell RNA-seq, data source: Supplementary Table 1) [17–19], (ii) *MAEL* mRNA expression in 32 types of cancer cell lines (Cancer Cell Line Encyclopedia [CCLE]) and 33 types of cancer tissues (The Cancer Genome Atlas [TCGA]) and its pan-cancer prognostic effects [20, 21], (iii) mRNA expression of the six transcripts of *MAEL* among 33 cancer types and its association with DNA methylation and copy number variations (CNVs) [22, 23], (iv) protein expression of MAEL in ccRCC in the HPA database [18] and mRNA expression of *MAEL* in patient-derived xenografts (GSE83820 and GSE36895) [24, 25], (v) associations of *MAEL* expression with clinicopathological features, mutations, gene expression, and prognosis in The Cancer Genome Atlas-Kidney Renal Clear Cell Carcinoma (TCGA-KIRC) cohort (n=522) [26] and the International Cancer Genome Consortium (ICGC)-Pan-cancer analysis of whole genomes (PCAWG) Renal Cell Carcinoma-Europe (RECA-EU) cohort (n=64) [27, 28], (vi) association between *MAEL* expression and sensitivities to VEGFR/mTOR inhibitors in the ccRCC cell lines of the Genomics of Drug Sensitivity in Cancer (GDSC, n=16) dataset [29] and patients with advanced ccRCC treated with first-line sunitinib (E-MTAB-3267, n=53) [30], and (vii) implications of *MAEL* expression in predicting the benefit from ICI-based therapies over VEGFR/mTOR inhibitors in the JAVELIN Renal 101 trial (phase III, avelumab+axitinib vs. sunitinib, n=726) and the CheckMate-025 trial (phase III, nivolumab vs. everolimus, n=250) [31].

The basic features of these clinical cohorts, including sample sizes, outcomes, clinical settings, the platforms of RNA-seq and immunohistochemical (IHC) staining of programmed cell death-ligand 1 (PD-L1), and treatment, are displayed in Supplementary Table 2. This report follows the Strengthening the Reporting of Observational Studies in Epidemiology (STROBE) and the REporting recommendations for tumour MARKer prognostic studies (REMARK) reporting guidelines.

### Genomic analysis

The genomic alterations of the TCGA-KIRC cohort were downloaded from the University of California Santa Cruz (UCSC) Xena database [32]. Tumor mutational burden (TMB) and intratumoral heterogeneity (ITH) were retrieved from the TCGA pan-cancer article [26]. The Catalogue Of Somatic Mutations In Cancer (COSMIC) database was used as a supplement to the TCGA-KIRC cohort for measuring the mutational rate of *MAEL* in ccRCCs. Silent mutations were excluded from our study. The genomic locations of the six transcripts of *MAEL* and their regulation regions (e.g., promoter and enhancer) were illustrated using the Ensembl [33].

### Transcriptomic analysis

*MAEL* expression in normal human tissue bulks and single cells was illustrated using the HPA (https://www.proteinatlas.org/ENSG00000143194-MAEL, for details, see Supplementary Methods) [17–19]. For tissue bulk RNA-seq, there are 107 samples of the nervous system (e.g., cerebral cortex) in the HPA dataset, and we used the median value to represent the *MAEL* expression in the nervous system. For single-cell RNA-seq, Uniform Manifold Approximation and Projection (UMAP) was used to visualize the different single-cell clusters, and the cell type of each cluster was determined by the expression of cell-type markers.

The expression of the six isoforms of *MAEL* in cancer tissue bulks and the prognostic effects of *MAEL* expression in the 33 cancer types of the TCGA database were analyzed using the Gene Expression Profiling Interactive Analysis 2 (GEPIA2, http://gepia2.cancer-pku.cn) [21]. The association between *MAEL* expression and its DNA methylation level was explored using the MEXPRESS (https://mexpress.be/index.html) [22, 23]. The level of transcriptomic data was measured by log_2_(transcripts per kilobase million [TPM]+1) in the present study.

Gene Ontology (GO) analysis was performed on the website (http://geneontology.org/) by using the annotation data set named Protein Analysis Through Evolutionary Relationships (PANTHER) GO-slim biological process [34, 35].

For Gene Set Enrichment Analysis (GSEA), the javaGSEA Desktop Application (GSEA 4.0.1) was used to investigate the gene signatures significantly enriched in the ccRCC samples with higher or lower *MAEL* expression (cut-off: median value) [36]. The normalized enrichment score (NES) is the primary statistic for assessing the enrichment of gene sets.

### Pharmacogenomic analysis

In total, 16 ccRCC cell lines with data on transcriptomics and sensitivities to anti-cancer agents were included for analysis. For each targeted agent, the half-maximal inhibitory concentration levels (IC_50_) of 16 ccRCC cell lines were scaled according to their geomean (formula: lg[*IC*_50_/geomean]). Two-way analysis of variance was used to assess the difference in sensitivities between the cell lines with high and low *MAEL* expression.

### Statistical analysis

To assess the between-group difference, we used (i) the Fisher exact test for categorical variables, (ii) the Mann-Whitney test, t test with Welch correction, the Kruskal-Wallis test, or one-way analysis of variance for continuous variables, and (iii) the Kaplan-Meier (KM) curves, the Log-rank test, and the Cox proportional-hazards regression model (hazard ratio [HR] and 95% confidence interval [CI]) for time-to-event variables. The variables with a p-value below 0.05 in the univariable Cox regression were included in the following multivariable Cox model. The spearman or Pearson correlation was used to test the correlations between continuous variables.

The statistical analyses were performed using IBM SPSS Statistics 22 or R 4.1.3. The nominal level of significance was set at 5%, and all 95% CIs were 2-sided unless otherwise specified. The adjusted P-value (Q-value) was calculated using the Benjamini-Hochberg method.

### Data availability

The authors declare that relevant data supporting the findings of this study are available within the paper and its Supplementary Files. Due to ethical and privacy concerns, we are unable to publish the patient-level data in our study, of which readers may contact the corresponding authors for the access for non-commercial purposes.

## RESULTS

### *MAEL* expression in normal tissues and single cells

**Figure 1:**
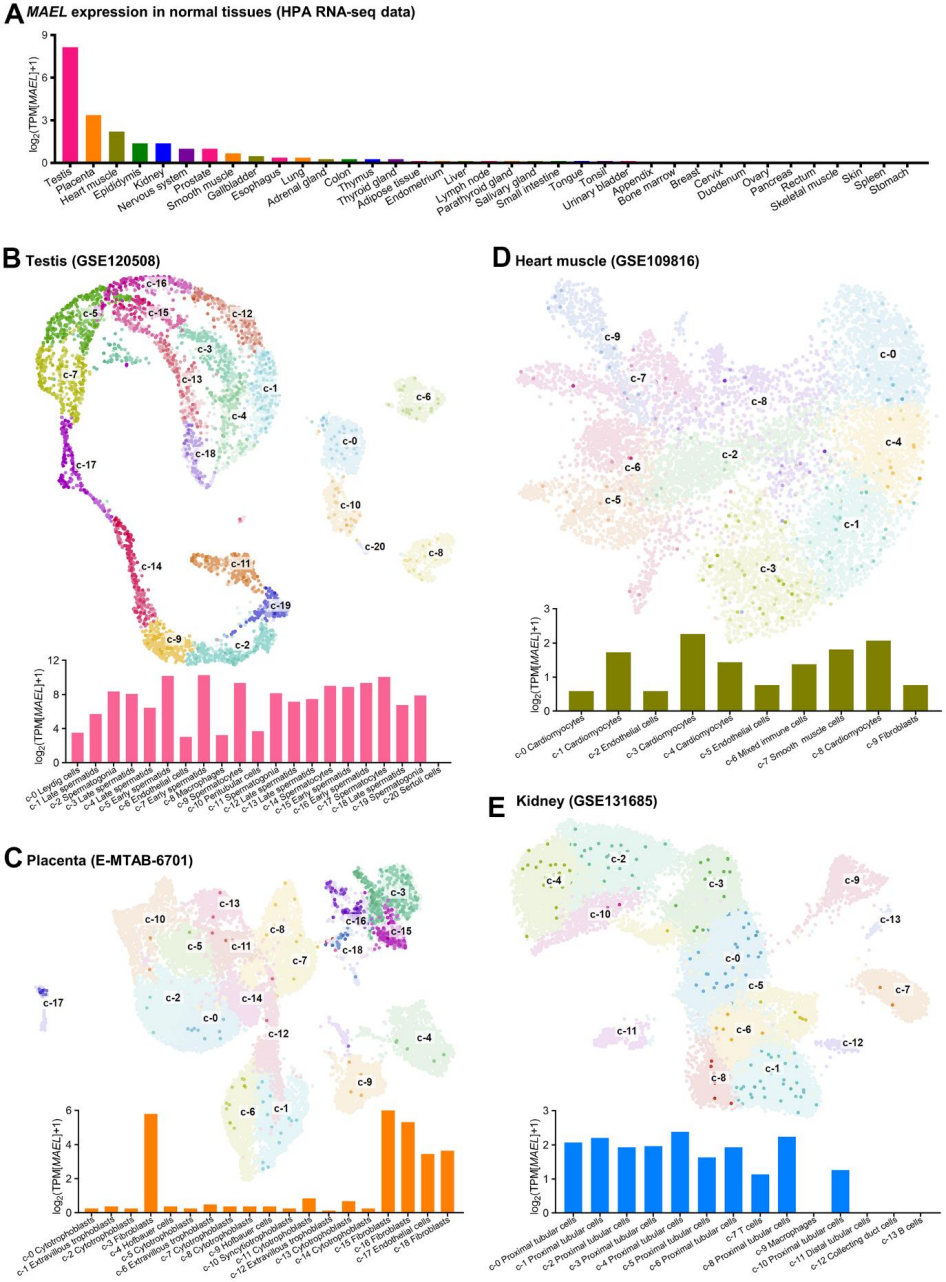
***MAEL* expression in normal tissues and single-cell clusters.** (**A**) *MAEL* expression in normal tissues (tissue bulk RNA-seq, Human Protein Atlas). (**B**–**E**) UMAP plots and *MAEL* expression in the single cell clusters (single cell RNA-seq) of testis (GSE120508, **B**), placenta (E-MTAB-6701, **C**), heart muscle (GSE109816, **D**), and kidney (GSE131685, **E**). The depth of the color of each point reflects the relative expression of *MAEL*. Abbreviations: TPM=transcripts per kilobase million, UMAP=Uniform Manifold Approximation and Projection.

A previous study observed that human MAEL expression was exclusive in testis rather than other tissues, including brain, heart, liver, lung, spleen, kidney, and ovary as determined by Northern blot (5). Here, we analyzed the RNA-seq data of 32 kinds of human tissues. Besides testis, relatively higher MAEL expression was revealed in placenta, heart muscle, epididymis, kidney, and nervous system (e.g., brain and spinal cord; Figure 1A and Supplementary Table 3).

We further analyzed the single-cell transcriptomes of 25 kinds of human tissues and peripheral blood mononuclear cells (PBMCs) to assess the expression level of *MAEL* in different cell types (Supplementary Table 4). As for the tissues with higher *MAEL* expression (testis, placenta, heart muscle, kidney, and brain), the UMAP plots and *MAEL* expression in each single cell cluster are shown in Figure 1B–1E and Supplementary Figure 1, and the corresponding mRNA expression of cell-type markers in different single cell type clusters are displayed in Supplementary Figure 2–6, respectively. In short, *MAEL* was expressed relatively higher in early spermatids, spermatocytes, spermatogonia, and late spermatids in testis (Figure 1B), fibroblasts and endothelial cells in placenta (Figure 1C), smooth muscle cells and cardiomyocytes in heart muscle (Figure 1D), proximal tubular cells in kidney (Figure 1E), and astrocyte, excitatory neurons, and oligodendrocyte precursor cells in brain (Supplementary Figure 1).

Of note, first, unlike the pattern in testis and placenta where *MAEL* was expressed “equivalently” in the single-cell clusters with higher *MAEL* expression (e.g., c-7 in testis and c-15 in placenta, Figure 1B, 1C), *MAEL* was expressed “sporadically” in the proximal tubular cells in kidney (Figure 1E), suggesting that *MAEL* might function in a certain subset of proximal tubular cells, probably relevant to the homeostasis of proximal tubule. Second, *MAEL* expression was extremely low in immune tissues (lymph node, spleen, bone marrow, and thymus, Figure 1A) and the immune cell clusters in other tissues and peripheral blood (Supplementary Table 4), raising the possibility that MAEL might not be involved in the maturation and activation of immune cells.

### *MAEL* in cancer tissues and cancer cell lines

**Figure 2:**
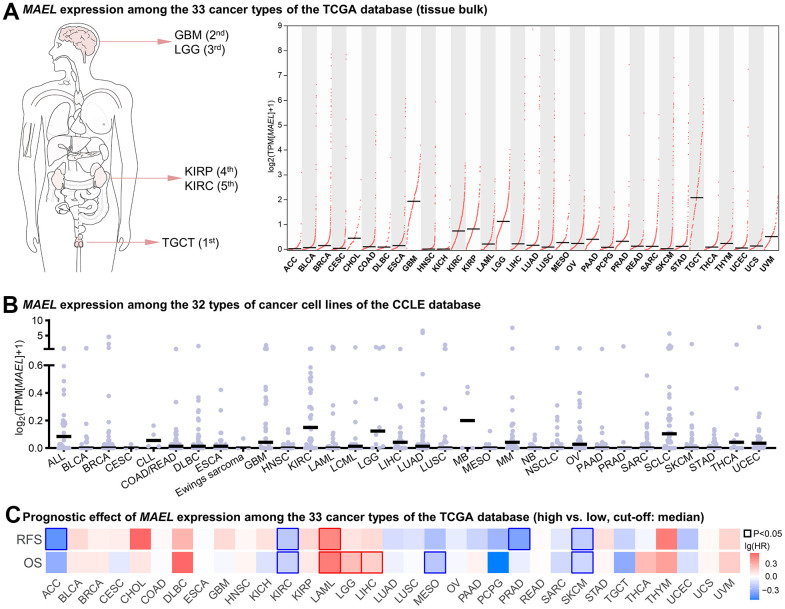
**Pan-cancer analysis of *MAEL*.** (**A**) *MAEL* expression among the 33 cancer types of the TCGA database (tissue bulk RNA-seq). (**B**) MAEL expression among the 32 types of cancer cell lines of the CCLE database. (**C**) Prognostic effect of *MAEL* expression among the 33 cancer types of the TCGA database (high vs. low, cut-off: median). Abbreviations: CCLE=Cancer Cell Line Encyclopedia, TCGA=The Cancer Genome Atlas.

Among the 33 cancer types in the TCGA database (abbreviations, see Supplementary Table 5), *MAEL* was expressed higher in TGCT, glioblastoma multiforme (GBM), brain lower-grade glioma (LGG), kidney renal papillary cell carcinoma (KIRP, also abbreviated as pRCC), and KIRC (also abbreviated as ccRCC; Figure 2A). These results were consistent with its expression in normal cell types. For instance, unlike pRCC and ccRCC originating from proximal tubular cells with high *MAEL* expression (Figure 1E), kidney chromophobe carcinoma (KICH) develops from distal tubular cells that did not express *MAEL* (Figure 1E) and had far lower expression of *MAEL* than KIRC and KIRP (Figure 2A). Similarly, in the CCLE database (abbreviations, see Supplementary Table 5), high *MAEL* expression was observed in nervous system tumors and KIRC (Figure 2B).

As for prognostic value, the RFS and OS of two subgroups divided by the median *MAEL* mRNA level were compared among the 33 cancer types in the TCGA database. Consistent prognostic effects for predicting both RFS and OS (P<0.05) were observed in KIRC, acute myeloid leukemia (LAML), and skin cutaneous melanoma (SKCM, Figure 2C).

Given the level of *MAEL* mRNA and its prognostic effect, MAEL may play a crucial role in KIRC, compared with other cancer types. We sought to further discover its linkages with clinicopathological features, DNA methylation, genomic alterations, pathway activation, drug sensitivity, and immunotherapy efficacy in clear cell renal cell carcinomas.

### Expression of the six transcripts of *MAEL* and its potential regulatory mechanisms in ccRCCs

**Figure 3:**
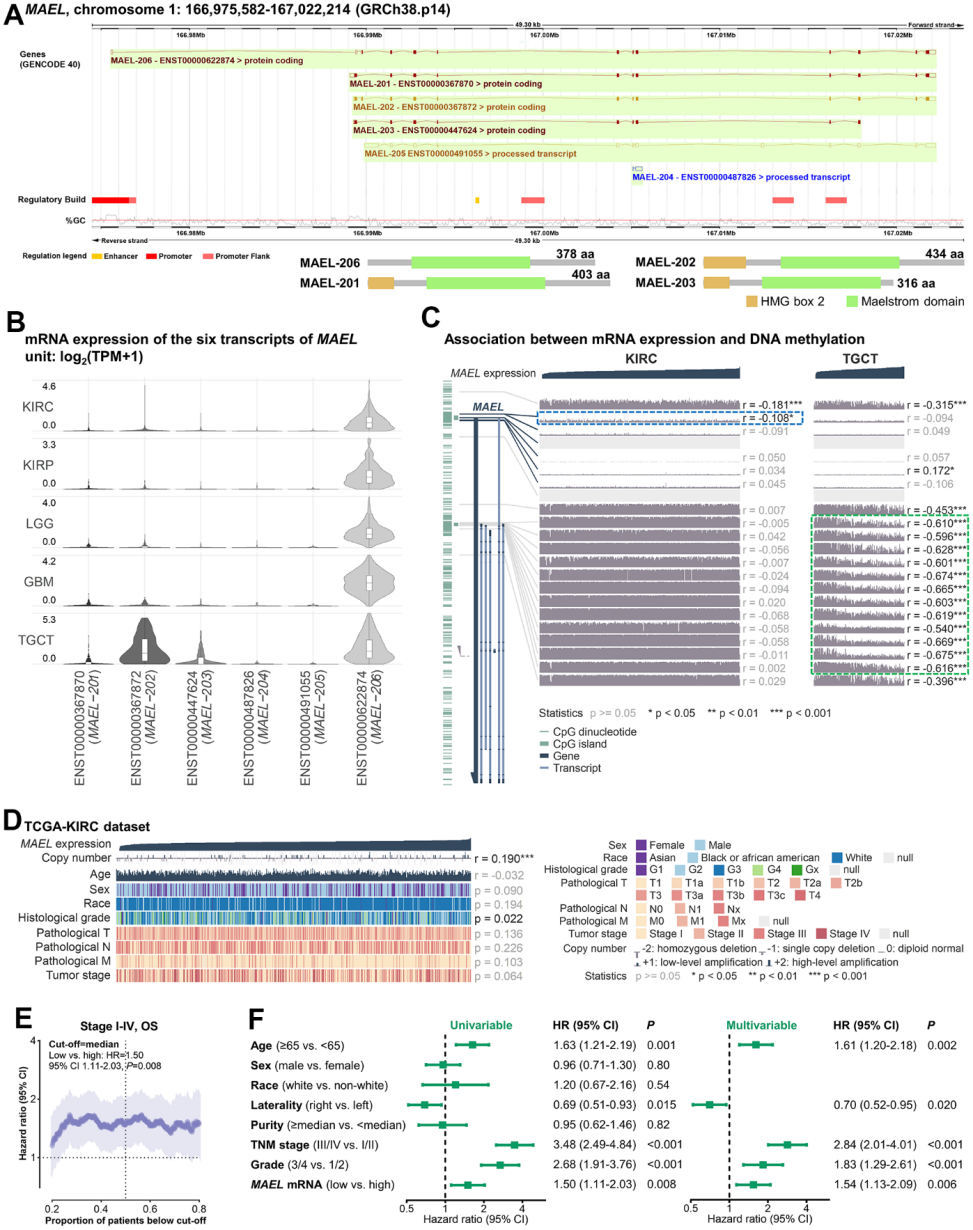
**Expression and prognostic effect of *MAEL* in ccRCC.** (**A**) Location of *MAEL* in human genome. (**B**) mRNA expression of the six transcripts of *MAEL* in the KIRCs, KIRPs, LGGs, GBMs, and TGCTs of the TCGA database. (**C**) Association between mRNA expression and DNA methylation in the KIRCs and TGCTs of the TCGA database. (**D**, **E**) Associations of *MAEL* expression with copy number, clinicopathological features (**D**) and overall survival (**E**) in the TCGA-KIRC cohort. (**F**) Univariable and multivariable analysis of the prognostic effect of *MAEL* expression in the TCGA-KIRC cohort. Abbreviations: ccRCC=clear cell renal cell carcinoma, GBM=glioblastoma multiforme, KIRC=Kidney Renal Clear Cell Carcinoma, KIRP=kidney renal papillary cell carcinoma, LGG=brain lower grade glioma, TCGA=The Cancer Genome Atlas, TGCT=testicular germ cell tumors.

*MAEL*, located in chromosome 1 (166,975,582-167,022,214), has six transcripts, of which *MAEL-204* and *MAEL-205* are processed transcripts, and the other four transcripts (*MAEL-206*, *MAEL-201*, *MAEL-202*, and *MAEL-203*) can be translated into proteins (Figure 3A; features of these isoforms are shown in Supplementary Table 6). In particular, MAEL-206 lacks the HMG domain compared with MAEL-201, 202, and 203 (Figure 3A).

The expression of these six transcripts in the 33 cancer types in the TCGA database is shown in Supplementary Figure 7. Among the five cancer types with the highest *MAEL* expression, TGCT had equivalent expression levels of *MAEL-206* and *MAEL-202*, while the *MAEL* expression in the other four cancer types was dominated by *MAEL-206* (>90%, Figure 3B), indicating the potential role of *MAEL-206* in brain and kidney tumors.

Given the distinct expression patterns of *MAEL* isoforms in KIRC and TGCT, we further explored the DNA methylation level of *MAEL* and its association with mRNA expression in these two cancers. First, in KIRCs where *MAEL* expression was dominated by *MAEL-206*, *MAEL* expression was negatively correlated with the methylation level of cg08348962 near the promoter of *MAEL-206* (P=0.049), while this association was non-significant in TGCTs (P=0.24; Figure 3C and Supplementary Table 7, marked in blue). Second, compared with KIRCs, TGCTs had higher *MAEL-202* expression and lower methylation levels in the regions near its promoters (Figure 3C, marked in green); the methylation levels of these regions were negatively correlated with *MAEL* expression in TCGTs instead of KIRCs (P<0.05 in TCGTs and P>0.20 in KIRCs; Supplementary Table 7, marked in green). These results indicate that DNA methylation may, in part, explain the distinct expression patterns of *MAEL* isoforms in human cancers.

In addition to DNA methylation, copy number and mutation may affect transcription. *MAEL* expression was positively correlated with copy number (P<0.001, Supplementary Table 7). No mutational event of *MAEL* was observed in the ccRCCs of the TCGA and the COSMIC databases, suggesting that its function in ccRCCs might be regulated by expression level instead of the mutant protein.

### Clinicopathological and prognostic correlates of *MAEL* in ccRCCs

Age, sex, race, and pathological TNM stage were not significantly associated with *MAEL* expression, while the samples with a poor histological grade had lower *MAEL* expression (P=0.022, Figure 3D and Supplementary Table 7). We further calculated the prognostic effect between *MAEL* expression and OS at each cut-off value ranging from 20^th^ to 80^th^ percentiles and observed that high *MAEL* expression trended to be associated with long OS at most cut-off values (Figure 3E). When the cut-off was empirically determined as the median value, the HR was 1.50 (low vs. high: 95% CI 1.11–2.03, P=0.008, Figure 3E). The prognostic effect of *MAEL* expression was independent of covariates including age, laterality, TNM stage, and histological grade (multivariable HR=1.54, 95% CI 1.13–2.09, P=0.006, Figure 3F). A similar association with RFS was also observed (univariable HR=1.43, 95% CI 1.00–2.04, P=0.050; multivariable HR=1.47, 95% CI 1.03–2.11, P=0.034; Supplementary Figure 8). In a small cohort retrieved from the ICGC-PCAWG RECA-EU database (n=64) [27, 28], we also observed a prognostic trend with similar HR (low vs. high: HR=1.58, 95% CI 0.70–3.58, P=0.28; Supplementary Figure 9).

MAEL protein expression in the cytoplasm of partial tumor cells was observed in two ccRCC samples of the HPA database using immunohistochemical staining (Supplementary Figure 10) [18]. This distribution profile is consistent with previous studies in hepatocellular carcinoma [14], ovarian cancer [15], bladder urothelial carcinoma [16], and colorectal adenocarcinoma [13]. Moreover, in the GSE83820 dataset including five ccRCC samples and their PDXs, compared to the primary grafts, the *MAEL* expression was increased at early passages (passage 1 [P1] vs. P0: P=0.014; P2 vs. P0: P=0.086) and tended to return to the baseline level at P4 (Supplementary Figure 11). A similar trend was observed in another PDX dataset (GSE36895, Supplementary Figure 11). These results indicate the stable expression of *MAEL* in ccRCC and suggest that MAEL might be involved in clonal evolution and/or immune escape during the early phase of xenograft development.

*MAEL* was expressed higher in ccRCCs than in normal kidneys (P=0.024, Supplementary Figure 12) indicating its oncogenic role in ccRCC, while higher *MAEL* expression was associated with a better prognosis. This observation might seem counterintuitive. However, different types of ccRCCs may depend on different oncogenes, and *MAEL*-dependent ccRCCs may progress more slowly than those dependent on other oncogenes, thus exhibiting a relatively better prognosis.

### Genomic, transcriptomic, and pharmacogenomic correlates of *MAEL* in ccRCCs

**Figure 4:**
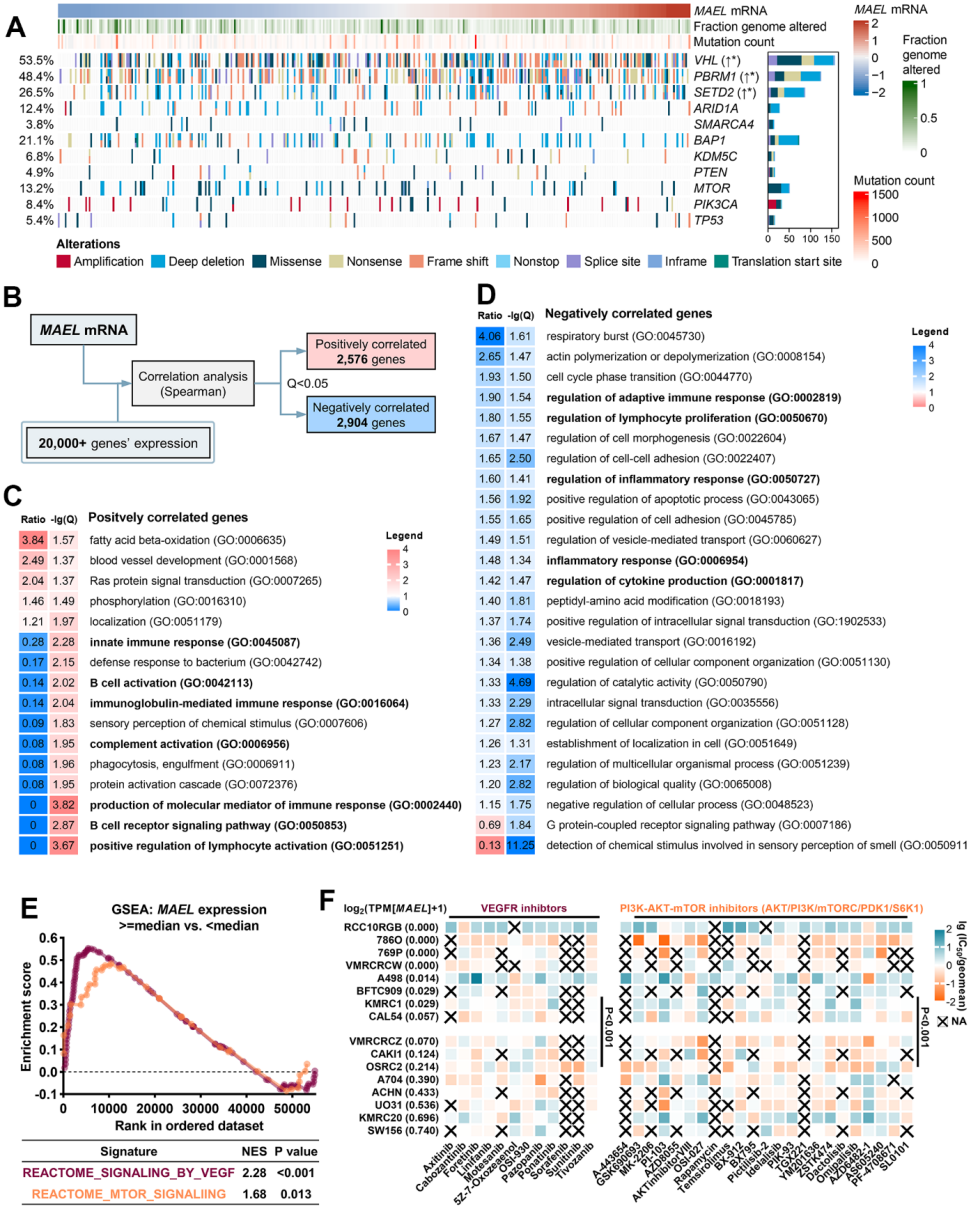
**Associations of the *MAEL* expression with genomic alterations, gene expression, and sensitivities to VEGFR and PI3K-AKT-mTOR inhibitors.** (**A**) Oncoprint illustrating the association between *MAEL* expression and genomic indices and alterations in the TCGA-KIRC cohort. (**B**) Diagram of identifying the genes with expression correlated with *MAEL* expression in the TCGA-KIRC cohort. (**C**, **D**) Gene Ontology results of the positively correlated genes (**C**) and negatively correlated genes (**D**). (**E**) Gene Set Enrichment Analysis results revealing the associations between *MAEL* expression (high vs. low, cut-off: median) and the enrichments of VEGF- and mTOR-related genes in the TCGA-KIRC cohort. (**F**) *MAEL* expression and its associations with the half-maximal inhibitory concentration levels in the 16 ccRCC cell lines. Abbreviations: IC_50_=half-maximal inhibitory concentration levels, TCGA-KIRC=The Cancer Genome Atlas-Kidney Renal Clear Cell Carcinoma.

In the TCGA-KIRC cohort, *MAEL* expression was not associated with mutational count (P=0.99) or fraction genome altered (P=0.27). As for commonly mutated genes, high *MAEL* expression was associated with the mutations in *VHL*, *PBRM1*, and *SETD2* (P<0.05, Figure 4A), suggesting its linkage with activated angiogenesis [37, 38].

We further analyzed the correlations between *MAEL* expression and other genes’ expression levels and identified 2,576 positively-correlated genes and 2,904 negatively-correlated genes (Figure 4B and Supplementary Table 8). The positively-correlated genes were enriched in the pathways concerning blood vessel development and Ras protein signal transduction (Q<0.05, Figure 4C) and excluded in the immune-related pathways about B cell, immunoglobulin-mediated immune response, lymphocyte activation, and complement activation (Q<0.05; Figure 4C, marked in bold). On the contrary, the negatively-correlated genes were enriched in the immune-related pathways relating to lymphocyte proliferation, inflammatory response, and cytokine (Q<0.05; Figure 4D, marked in bold).

Inhibitors of vascular endothelial growth factor receptor (VEGFR, e.g., sunitinib and axitinib) and mTOR (e.g., everolimus) exhibit anti-tumor activity in ccRCCs [39–41], largely due to the activation of angiogenesis and the PI3K-AKT-mTOR signaling [42–45]. Compared with the ccRCCs with low *MAEL* expression (below median), VEGF and mTOR signatures were enriched in those with high *MAEL* expression (VEGF: NES=2.28, P<0.001; mTOR: NES=1.68, P=0.013; Figure 4E). Among the 16 ccRCC cell lines in the CCLE database, the IC_50_ values of VEGFR and PI3K-AKT-mTOR inhibitors were lower in those with high *MAEL* expression than those with low *MAEL* expression (above median; P<0.001, Figure 4F and Supplementary Table 9). In the E-MTAB-3267 cohort including 53 patients with advanced ccRCC [30], high *MAEL* expression trended to be linked with favorable PFS on first-line sunitinib (high vs. low: HR=0.56, 95% CI 0.30–1.06, P=0.064, Supplementary Figure 13).

Taken together, *MAEL* expression was associated with inactivated anti-tumor immunity, activated pathways concerning VEGFR and PI3K-AKT-mTOR, and sensitivities to VEGFR/PI3K-AKT-mTOR inhibitors in ccRCCs.

### *MAEL* expression predicts the benefits from ICI-based therapies over VEGFR/mTOR inhibitors in advanced/metastatic ccRCCs

We further investigated the association of *MAEL* expression with the benefit from ICI-based therapies over VEGFR/mTOR inhibitors in two large phase III trials, the JAVELIN Renal 101 (avelumab plus axitinib vs. sunitinib) and the CheckMate-025 (nivolumab vs. everolimus).

**Figure 5:**
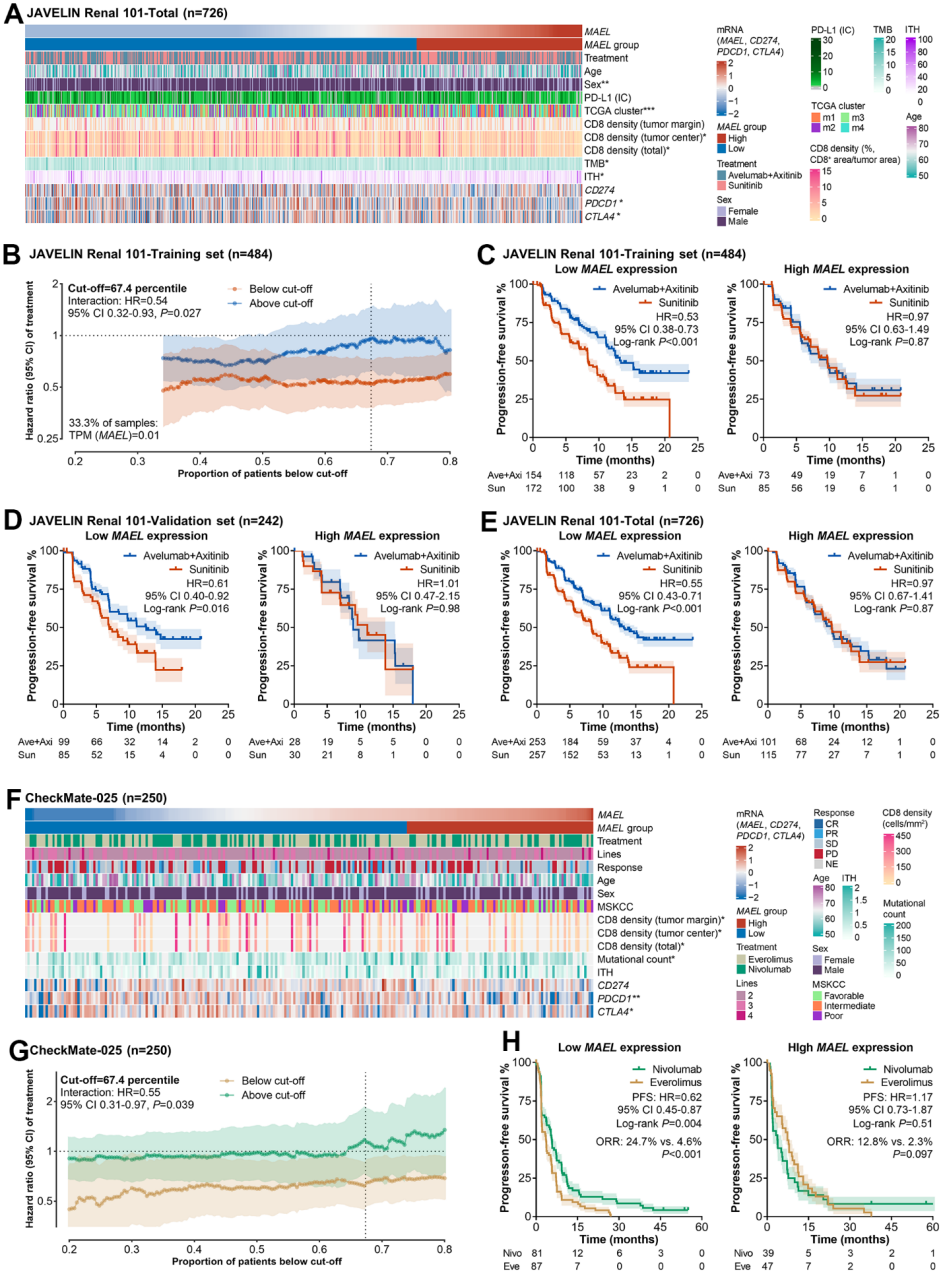
***MAEL* expression predicts the benefit from ICI-based immunotherapies over VEGFR/mTOR inhibitors in advanced/metastatic ccRCCs.** (**A**) Heatmap illustrating *MAEL* expression and clinicopathological features of the JAVELIN Renal 101 cohort. (**B**) The associations of the cut-off value with the treatment effect in the above- and the below-cut-off groups in the training set of the JAVELIN Renal 101 cohort. (**C**–**E**) The treatment effect (avelumab plus axitinib vs. sunitinib) in the low and the high *MAEL* expression groups in the training set (**C**), the validation set (**D**), and the total set (**E**) of the JAVELIN Renal 101 cohort. (**F**) Heatmap illustrating *MAEL* expression and clinicopathological features of the CheckMate-025 cohort. (**G**) The associations of the cut-off value with the treatment effect in the above- and the below-cut-off groups of the CheckMate-025 cohort. (**H**) The treatment effect (nivolumab vs. everolimus) in the low and the high *MAEL* expression groups of the CheckMate-025 cohort. Abbreviations: CI=confidence interval, CR=complete response, HR=hazard ratio, IC=immune cell, ITH=intratumoral heterogeneity, NE=not evaluable, ORR=objective response rate, PD=progressive disease, PD-L1=programmed cell death-ligand 1, PR=partial response, SD=stable disease, TCGA=The Cancer Genome Atlas, TMB=tumor mutational burden.

First, the 726 advanced/metastatic ccRCC patients with available RNA-seq data in the JAVELIN Renal 101 trial (clinicopathological features, see Figure 5A) were randomly separated into a training set (n=484) and a validation set (n=242) with a ratio of 2:1. The difference in the association of a biomarker with survival across treatment arms is the essential proof of its predictive utility [46]. In the training set, for each cut-off value ranging from 20^th^ to 80^th^ percentiles, we calculated the treatment effect in the below cut-off and the above cut-off subgroups. The treatment effect was larger in the low *MAEL* group than the high *MAEL* group at all cut-offs (Figure 5B). The difference in treatment effect between these two subgroups reached its maximum at the cut-off of 67.4^th^ percentile (interaction HR=0.54, 95% CI 0.32–0.93, P=0.027, Figure 5B). At this cut-off, the benefit from avelumab plus axitinib over sunitinib was considerable in the low *MAEL* expression group (HR=0.53, 95% CI 0.38–0.73, P<0.001) while negligible in the high *MAEL* expression group (HR=0.97, 95% CI 0.63–1.49, P=0.87, Figure 5C). Comparable results were observed in the validation set (low *MAEL* expression group: HR=0.61, 95% CI 0.40–0.92, P=0.016; high *MAEL* expression group: HR=1.01, 95% CI 0.47–2.15, P=0.98, Figure 5D).

In the total set of all 726 patients, the interaction effect between *MAEL* expression and treatment effect was significant (interaction HR=0.56, 95% CI 0.36–0.88, P=0.012, Figure 5E). In the multivariable analysis using the data provided by the JAVELIN Renal 101 researchers, the interaction effect remained significant (multivariable interaction HR=0.58, 95% CI 0.37–0.91, P=0.019, Table 1). Compared with the low *MAEL* expression group, the high *MAEL* expression group had more females (P=0.001) and the m1/4 cluster defined by the TCGA Research Network study (P<0.001) [47], lower CD8 densities in tumor center (P=0.044) and total area (P=0.040), higher TMB (P=0.028) and ITH (P=0.022), and lower expression of *PDCD1* (P=0.010) and *CTLA4* (P=0.038, Figure 5A). Of note, *MAEL* expression was not associated with the *CD274* mRNA expression in tissue bulk (P=0.46) and the PD-L1 protein expression on immune cells (P=0.57, Figure 5A), indicating the irrelevance between the predictive utility of MAEL and PD-L1 expression.

In the CheckMate-025 trial involving 250 advanced/metastatic ccRCC patients with available RNA-seq data (Figure 5F), the curves of treatment effect in the “below cut-off” and “above cut-off” subgroups are shown in Figure 5G. The treatment effect was larger in the low *MAEL* group than the high *MAEL* group at all cut-offs (Figure 5G). At the cut-off (67.4^th^ percentile) derived from the training set of the JAVELIN Renal 101 cohort, the interaction effect was significant (HR=0.55, 95% CI 0.37–0.97, P=0.039, Figure 5G). Nivolumab delivered a significantly higher ORR and longer PFS than everolimus in the low *MAEL* expression subgroup (ORR: 24.7% vs. 4.6%, P<0.001; PFS: HR=0.62, 95% CI 0.45–0.87, P=0.004), but not in the high Notch-score subgroup (ORR: 12.8% vs. 2.3%, P=0.097; PFS: HR=1.17, 95% CI 0.73–1.87, P=0.51; Figure 5H). The interaction effect remained significant in the multivariable model (multivariable interaction HR=0.51, 95% CI 0.29–0.91, P=0.023, Table 1). High *MAEL* expression was associated with few CD8^+^ T cells in tumor center (P=0.040), tumor margin (P=0.016), and total area (P=0.026), high TMB (P=0.013), and low expression of *PDCD1* (P=0.003) and *CTLA4* (P=0.049) rather than *CD274* (P=0.12, Figure 5F).

**Table 1:** Predictive effect of the *MAEL* expression in multivariable models.

**Parameter**	**JAVELIN Renal 101: progression-free survival**			
	**Univariable analysis**		**Multivariable analysis**	
	**HR (95% CI)**	**P-value**	**HR (95% CI)**	**P-value**
Age (≥65 vs. <65)	0.74 (0.60-0.93)	0.009	0.77 (0.61-0.96)	0.018
Sex (male vs. female)	0.90 (0.71-1.14)	0.38		
PD-L1 mRNA (≥median vs. <median)	0.91 (0.74-1.12)	0.37		
PD-L1 IC score (dummy variable)		0.43		
1-9 vs. 0	1.14 (0.90-1.44)	0.28		
>=10 vs. 0	1.23 (0.87-1.74)	0.25		
CD8^+^ density (≥median vs. <median)				
Tumor center	0.95 (0.77-1.18)	0.67		
Tumor margin	1.03 (0.76-1.41)	0.83		
Total	0.93 (0.75-1.16)	0.52		
TMB (≥median vs. <median)	0.95 (0.76-1.17)	0.61		
ITH (≥median vs. <median)	1.15 (0.93-1.42)	0.20		
TCGA subtype (dummy variable)		0.013		0.024
2 vs. 1	1.01 (0.73-1.40)	0.93	1.07 (0.77-1.48)	0.70
3 vs. 1	1.52 (1.15-2.01)	0.004	1.53 (1.15-2.03)	0.003
4 vs. 1	1.20 (0.87-1.65)	0.26	1.16 (0.85-1.60)	0.35
NA vs. 1	0.82 (0.38-1.77)	0.61	0.93 (0.43-2.04)	0.86
Treatment (avelumab+axitinib vs. sunitinib)	0.98 (0.67-1.42)	0.91	0.98 (0.67-1.43)	0.92
*MAEL* expression (low vs. high)	1.23 (0.91-1.67)	0.19	1.16 (0.85-1.58)	0.34
**Interaction between treatment and *MAEL* expression**	**0.56 (0.36-0.88)**	**0.012**	**0.58 (0.37-0.91)**	**0.019**
**Parameter**	**CheckMate-009/010/025: progression-free survival**			
	**Univariable analysis**		**Multivariable analysis**	
	**HR (95% CI)**	**P-value**	**HR (95% CI)**	**P-value**
Age (≥65 vs. <65)	0.91 (0.69-1.19)	0.50		
Sex (male vs. female)	1.15 (0.85-1.55)	0.38		
PD-L1 mRNA (≥median vs. <median)	1.00 (0.77-1.30)	0.99		
MSKCC risk (dummy variable)		0.019		0.010
Intermediate vs. favorable	1.14 (0.84-1.53)	0.40	1.21 (0.90-1.64)	0.21
Poor vs. favorable	1.69 (1.17-2.46)	0.006	1.78 (1.22-2.59)	0.003
CD8+ density				
Tumor center (≥median vs. <median)	1.11 (0.65-1.89)	0.70		
Tumor margin (≥median vs. <median)	1.21 (0.72-2.05)	0.48		
Total (≥median vs. <median)	1.11 (0.65-1.89)	0.71		
Treatment lines (dummy variable)		0.73		
3 vs. 2	1.11 (0.83-1.49)	0.47		
4 vs. 2	1.14 (0.63-2.05)	0.67		
TMB (≥median vs. <median)	1.09 (0.79-1.51)	0.58		
ITH (≥median vs. <median)	1.05 (0.75-1.46)	0.79		
Treatment (nivolumab vs. everolimus)	1.15 (0.72-1.83)	0.57	1.17 (0.73-1.86)	0.51
*MAEL* expression (low vs. high)	1.62 (1.10-2.39)	0.014	1.66 (1.13-2.45)	0.011
**Interaction between treatment and *MAEL* expression**	**0.55 (0.31-0.97)**	**0.039**	**0.51 (0.29-0.91)**	**0.023**

Abbreviations: CI, confidence interval; HR, hazard ratio; IC, immune cell; ITH, intratumoral heterogeneity; MSKCC, memorial sloan-kettering cancer center; NA, not applicable; PD-L1, programmed cell death-ligand 1; PFS, progression-free survival; TMB, tumor mutational burden.

**Figure 6:**
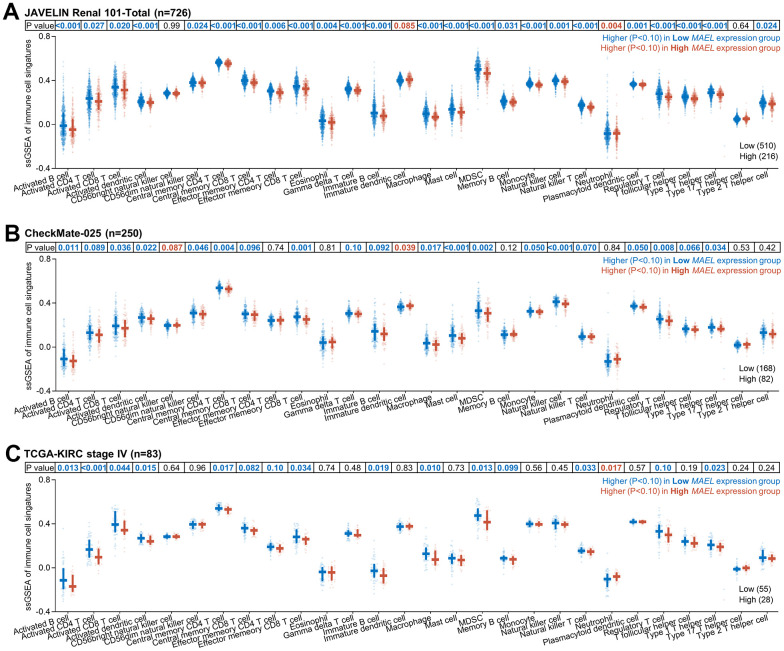
**Association between *MAEL* expression and immune cell signatures in advanced/metastatic ccRCCs.** (**A**–**C**) Association between *MAEL* expression and immune cell signatures in the JAVELIN Renal 101 cohort (**A**), the CheckMate-025 cohort (**B**), and the stage IV ccRCC patients in the TCGA-KIRC cohort (**C**). Abbreviations: MDSC=myeloid-derived suppressor cell, TCGA-KIRC=The Cancer Genome Atlas-Kidney Renal Clear Cell Carcinoma.

We further analyzed the association between *MAEL* expression and immune cell signatures in advanced/metastatic ccRCCs. In the three cohorts (JAVELIN, CheckMate, and TCGA), high *MAEL* expression was consistently linked with low levels of the signatures concerning activated B, CD4^+^ T, CD8^+^ T, and dendritic cells, central memory CD4^+^ and CD8^+^ T cells, effector memory CD8^+^ T cell, immature B cell, macrophage, MDSC, natural killer T cell, regulatory T cell, and type I T helper cell (P<0.10, detailed statistics, see Figure 6A–6C), indicating an anti-inflammatory microenvironment.

Taken together, high *MAEL* expression was identified as an independent indicator of poor benefits from ICI-based therapies over VEGFR/mTOR inhibitors in advanced ccRCCs, potentially mediated by tumor-infiltrating immune cells and the expression of PD-1 and CTLA-4 rather than PD-L1.

## DISCUSSION

In this study, we first delineated the expression landscape of *MAEL* in human normal tissues and cancers. Given that *MAEL* was highly expressed and was associated with both RFS and OS in ccRCCs, we investigated its implications in this cancer type in depth. High *MAEL* expression was associated with anti-inflammatory TIME, enhanced VEGFR and mTOR activities, and high sensitivities to VEGFR/PI3K-AKT-mTOR inhibitors. In the two clinical trials, the PFS benefits from ICI-based therapies over VEGFR/mTOR inhibitors were minimal in the high *MAEL* expression group but significant in the low *MAEL* expression group.

In all tumors except TGCT, MAEL is dominated by the MAEL-206 isoform, which lacks the HMG domain in the N terminal compared to the full-length MAEL. This expression pattern may be partly controlled by the methylation levels of the promoters of different isoforms. So far, nearly all published MAEL-associated cancer studies have been conducted in non-TGCT cell lines using plasmids carrying the full-length human *MAEL* cDNA. It might be more appropriate to carefully discern the function of each MAEL isoform in cell line and animal studies.

*MAEL*, as a potential oncogene, was expressed higher in ccRCCs than in normal kidneys, while high *MAEL* expression was identified as an independent indicator of favorable prognosis. This observation might seem counterintuitive. However, different types of ccRCCs may depend on different oncogenes, and *MAEL*-dependent ccRCCs may progress more slowly than those dependent on other oncogenes, thus exhibiting a relatively better prognosis. This oncogenic and prognostic pattern was also observed in other SG genes associated with *MAEL*, such as G3BP stress granule assembly factor 1/2 (*G3BP1/2*) [21, 48], suggesting that the MAEL/SG-dependent ccRCCs might progress more slowly compared with other ccRCCs.

In ccRCC, high *MAEL* expression was associated with VEGFR/mTOR activation and an anti-inflammatory TIME, which can explain the high sensitivities to VEGFR/PI3K-AKT-mTOR inhibitors and the poor benefit from ICI-based therapies over VEGFR/mTOR inhibitors. The associations of MAEL with AKT activation and a suppressive TIME have been disclosed in the cell lines of hepatocellular carcinoma and esophageal squamous cell carcinoma [11, 14]. The interaction between a biomarker and treatment effect (difference in the association of a biomarker with survival across treatment arms) is the essential proof of its predictive utility [46]. The interaction effects between *MAEL* expression and treatment choice in the two phase III trials were both significant, implying that, compared to VEGFR/mTOR inhibitors, ICI-based immunotherapies might be recommended for the ccRCCs with low *MAEL* expression. Due to the lack of patient-level data, it is not available to validate our results in other trials, e.g., CheckMate-214 and IMmotion151 [49, 50].

As for limitations, first, the molecular correlates of *MAEL* were analyzed using bioinformatic methods in our study. Biological validation using cell lines and xenograft models is warranted. Here, *MAEL* expression and its association with the sensitivity of VEGFR/mTOR inhibitors were observed in ccRCC cell lines. In addition, according to the single-cell data, *MAEL* expression was undetectable in most of the peripheral blood mononuclear cells and the endothelial cells and fibroblasts in abdominal organs, suggesting that the results derived from tissue-bulk RNA data may reflect the characteristics of MAEL in ccRCC tumor cells instead of other cells, including endothelial cells, fibroblasts, and tumor-infiltrating immune cells. Second, the retrospective setting of our study may introduce biases, which can be minimized by the context of large randomized phase III trials and the implementation of multivariable analysis and independent validation. Third, the raw RNA-seq data from the public datasets are hard to obtain, so it’s impossible to comprehensively analyzed the predictive utility of each *MAEL* transcript. Fortunately, the *MAEL* expression in ccRCCs was dominated by MAEL-206 (proportion>90%) and therefore our results based on the total *MAEL* expression can effectively reflect the effects of the dominant MAEL-202 in ccRCCs. Fourth, immune cell infiltration was estimated by ssGSEA in this study. Multiplex immunofluorescence of immune cell markers and MAEL in ccRCC samples would be beneficial for exploring the differences in tumor-infiltrating immune cells around *MAEL*-expressing and *MAEL*-non-expressing tumor cells. Fifth, the ICI regimens analyzed are avelumab plus axitinib and nivolumab monotherapy, which may represent anti-PD-(L)1 plus VEGFR inhibitor and anti-PD-(L)1 monotherapy, respectively. The combination of anti-PD-(L)1 and anti-CTLA-4 (e.g., CheckMate-214) was not included in our study due to the lack of patient-level data. Despite this, a negative association between *MAEL* expression and *CTLA-4* expression was observed in the two trials, suggesting the potential predictive utility of low *MAEL* expression for a large benefit from combination immunotherapy including anti-CTLA-4 over monotherapies of VEGFR/mTOR inhibitors.

To our knowledge, this is the first comprehensive analysis of *MAEL* in human cancers. High *MAEL* expression was observed in TGCT, glioma, pRCC, and ccRCC. Especially in ccRCC, *MAEL* is a biologically and clinically significant determinant with potential for prognostication after nephrectomy and patient selection for VEGFR/mTOR inhibitors and ICI-based immunotherapies. ICIs provide limited advantages and might not be strongly recommended for ccRCCs with high *MAEL* expression, by which the cost-effectiveness of treatments in ccRCCs may be potentially improved.

## Supplementary Material

Supplementary Methods

Supplementary Figures

Supplementary Tables 1 and 2

Supplementary Table 3

Supplementary Table 4

Supplementary Tables 5-7

Supplementary Table 8

Supplementary Table 9
