# Supplementary material for: MAEL in human cancers and implications in prognostication and predicting benefit from immunotherapy over VEGFR/mTOR inhibitors in clear cell renal cell carcinoma: a bioinformatic analysis
Source: Aging (Albany NY). 2024 Jan 31;16(3):2090–122. doi: 10.18632/aging.205470 (PMC10911358; doi:10.18632/aging.205470)
Supplement: Supplementary Methods [file aging-16-205470-s001.pdf]

SUPPLEMENTARY METHODS

Human protein atlas (HPA) RNA-seq data of normal tissue and blood samples

For normal tissue and blood samples, specimens were collected with consent from patients and all samples were anonymized in accordance with approval from the local ethics committee (ref #2011/473 and ref #2015/1552-32) and Swedish rules and legislation. All tissues were collected from the Uppsala Biobank and RNA samples were extracted from frozen tissue sections. Blood samples were enriched for PBMC and granulocytes, labeled with antibodies and separated into subpopulation by flow sorting. mRNA sequencing was performed on Illumina HiSeq2000 and 2500 machines (Illumina, San Diego, CA, USA) using the standard Illumina RNA-seq protocol with a read length of 2x100 bases. Blood cells mRNA sequencing was performed on an Illumina NovaSeq 6000 System in four S4 lanes with a read length of 2x150 bases. Transcript abundance estimation was performed using Kallisto v0.46.2. The 18 blood cell types are classified into six different lineages including B-cells, T-cells, NK-cells, monocytes, granulocytes, and dendritic cells.

The HPA Human brain sample set contains 1324 samples of >200 regions of the human brain. The analysis is a collaboration with Human Brain Tissue Bank (HBTB; Semmelweis University, Budapest) in accordance with approval from the Committee of Science and Research Ethic of the Ministry of Health Hungary (ETT TUKEB: 189/KO/02.6008/2002/ETT) and the Semmelweis University Regional Committee of Science and Research Ethic (No. 32/1992/TUKEB) to remove human brain tissue samples, collect, store and use them for research. Samples were collected by Prof. Palkovits and RNA was extracted from frozen brain punches. The human prefrontal cortex dataset includes 165 samples from 3 male and 3 female donors providing a detailed overview of protein expression in 17 subregions of the prefrontal cortex and 3 reference cortical regions was analyzed using the Illumina sequencing platform, all other samples were analyzed using the MGI DNBSEQ-T7 platform.

For more detail, please see https://www.proteinatlas.org/about/assays+annotation#hpa_rna.

Single cell RNA-seq data

The single cell RNA sequencing dataset is based on meta-analysis of literature on single cell RNA sequencing and single cell databases that include healthy human tissue. To avoid technical bias and to ensure that the single cell dataset can best represent the corresponding tissue, the following data selection criteria were applied: (1) Single cell transcriptomic datasets were limited to those based on the Chromium single cell gene expression platform from 10X Genomics (version 2 or 3); (2) Single cell RNA sequencing was performed on single cell suspension from tissues without pre-enrichment of cell types; (3) Only studies with >4,000 cells and 20 million read counts were included; (4) Only dataset whose pseudo-bulk transcriptomic expression profile is highly correlated with the transcriptomic expression profile of the corresponding HPA tissue bulk sample were included. It should be noted that exceptions were made for lung (~7.3 million reads), pancreas (3,719 cells) and rectum (3,898 cells) to include various cell types in the analysis.

In total, single cell transcriptomics data for 25 tissues and peripheral blood mononuclear cells (PBMCs) were analyzed. These datasets were respectively retrieved from the Single Cell Expression Atlas, the Human Cell Atlas, the Gene Expression Omnibus, the Allen Brain Map, and the European Genome-phenome Archive. The complete list of references is shown in Supplementary Table 2.

For each of the single cell transcriptomics datasets, the quantified raw sequencing data were downloaded from the corresponding depository database based on the accession number provided by the corresponding study in the available format (total cells, read, and feature counts, or count tables). Unfiltered data were used as input for downstream analysis with an in-house pipeline using Scanpy (version 1.4.4.post1) in Python 3.7.3 for the 13 tissues and PBMC published in HPA v20 and Scanpy (version 1.7.2) in Python 3.8.5 for the 12 tissues published in HPA v21. In the pipeline, the data were filtered using two criteria: a cell is considered as valid if at least 200 genes are detected and a gene is considered as valid if it is expressed in at least 10% of the cells. Specially, in the HPA v21, tissues which contain more than 10,000 cells used 1000 cells as their cutoff. Subsequently, the cell counts were normalized to have a total count per cell of 10000. The valid cells were then clustered using Louvain clustering function within Single-Cell Analysis in Python (Scanpy). Default values of parameters were used in clustering. More in detail, the features of cells were projected into a PCA space with 50 components using UMAP, and a k-nearest neighbours (KNN) graph was generated. 15 neighbours were used in the network for Louvain, while the resolution of clustering was set as 1.0. The total read counts for all genes in each cluster was calculated by adding up the read counts of each gene in all cells belonging to the corresponding cluster. Finally, the

read counts were normalized to transcripts per million protein coding genes (pTPM) for each of the single cell clusters. When calculating the expression profile for pseudo-bulk samples based on single cell transcriptomics, we added the read counts for all genes from all cells of the sample, and normalized it to pTPM in the same way as for the cluster ones.

Each of the 444 different cell type clusters were manually annotated based on an extensive survey of >500 well-known tissue and cell type-specific markers, including both markers from the original publications, and additional markers used in pathology diagnostics. For each cluster, one main cell type was chosen by taking into consideration the expression of different markers. For a few clusters, no main cell type could be selected, and these clusters were not used for gene classification. The most relevant markers are presented in a heatmap on the Cell Type Atlas, in order to clarify cluster annotation to visitors.

The cell type dendrogram presented on the Single Cell Type section shows the relationship between the single cell types based on genome-wide expression. The dendrogram is based on agglomerative clustering of 1 - Spearman's rho between cell types using Ward's criterion. The dendrogram was then transformed into a hierarchical graph, and link distances were normalized to emphasize graph connections rather than link distances. Link width is proportional to the distance from the root, and links are colored according to cell type group if only one cell type group is present among connected leaves.

For more detail, please see https://www.proteinatlas.org/about/assays+annotation#singlecell_rna.
