# Supplementary material for: MAEL in human cancers and implications in prognostication and predicting benefit from immunotherapy over VEGFR/mTOR inhibitors in clear cell renal cell carcinoma: a bioinformatic analysis
Source: Aging (Albany NY). 2024 Jan 31;16(3):2090–122. doi: 10.18632/aging.205470 (PMC10911358; doi:10.18632/aging.205470)
Supplement: Supplementary Figures [file aging-16-205470-s002.pdf]

SUPPLEMENTARY FIGURES

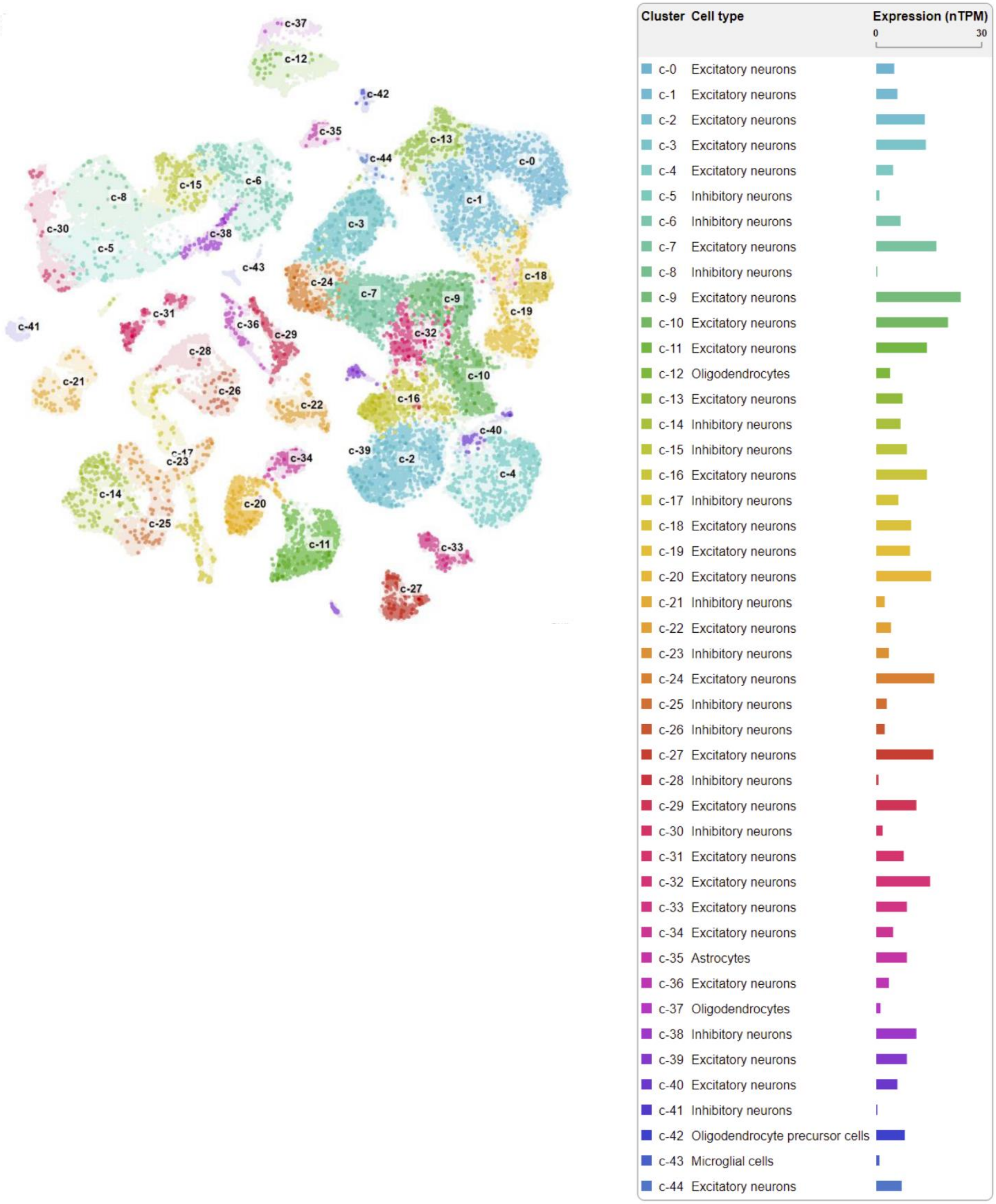

Supplementary Figure 1. UMAP plot and *MAEL* expression in the single cell clusters of normal brain (Allen brain map).

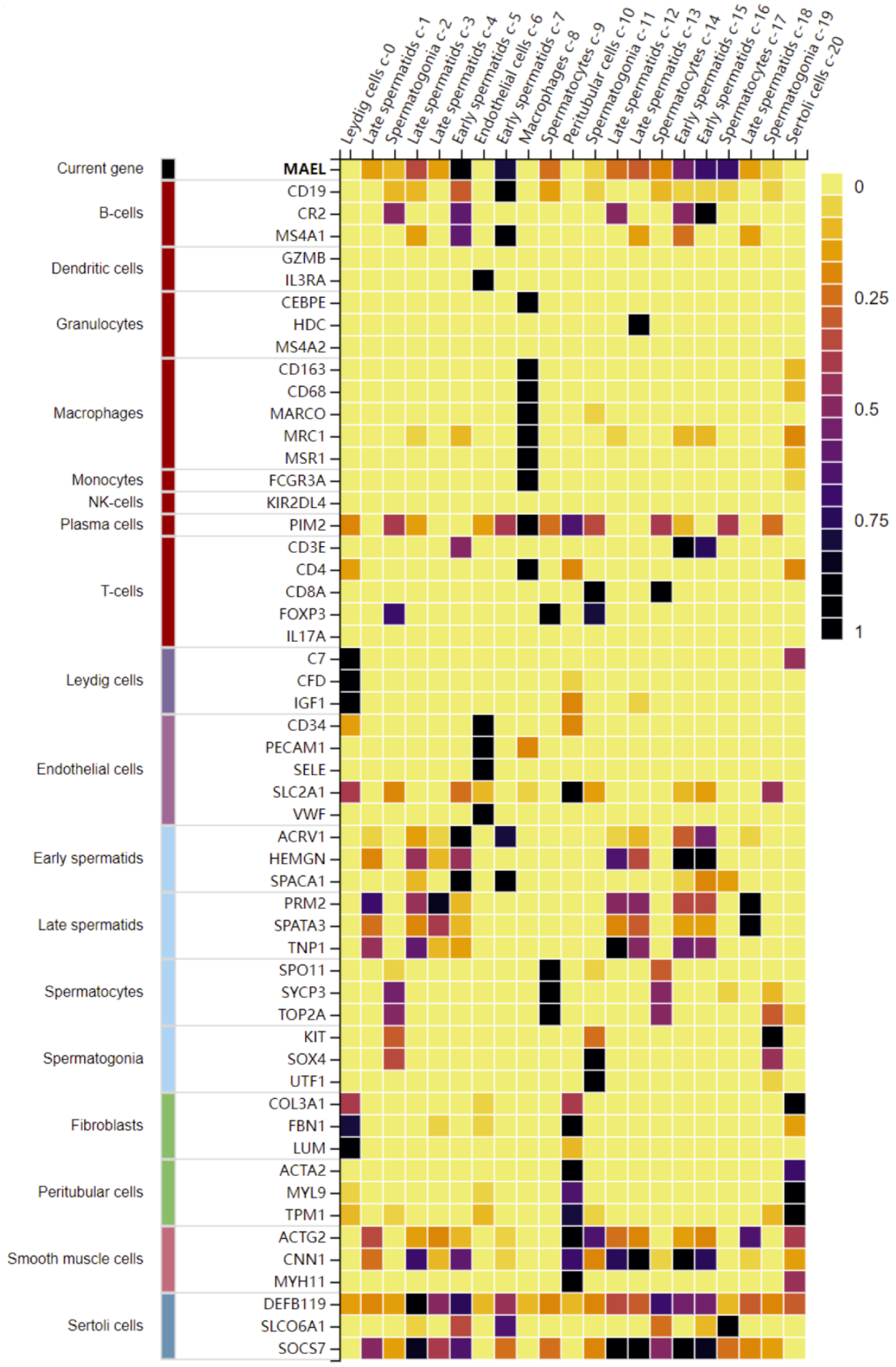

Supplementary Figure 2. mRNA expression of *MAEL* and cell type markers in different single cell type clusters of normal testis (GSE120508). Color legend: fraction of the highest expression.

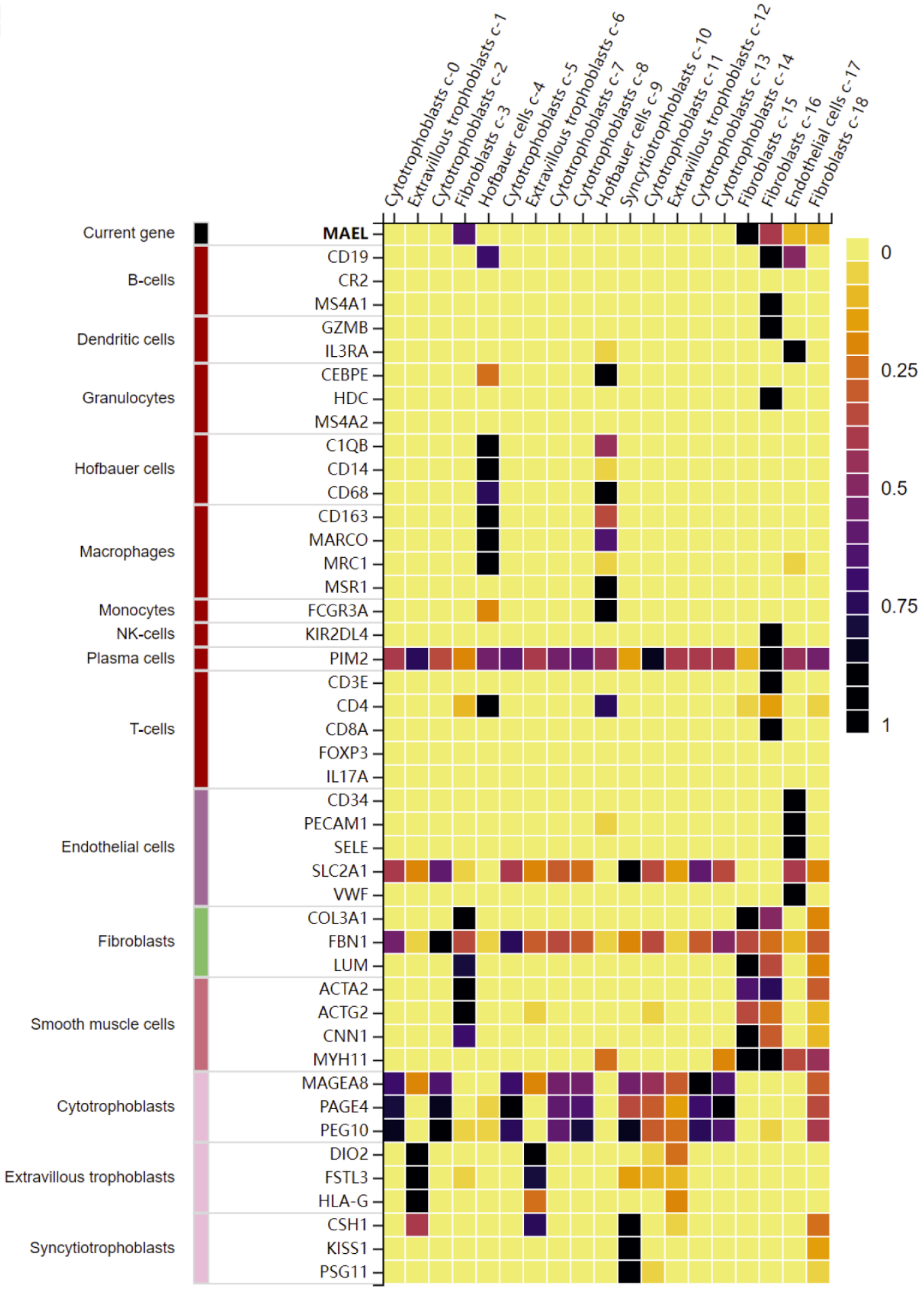

Supplementary Figure 3. mRNA expression of *MAEL* and cell type markers in different single cell type clusters of normal placenta (E-MTAB-6701). Color legend: fraction of the highest expression.

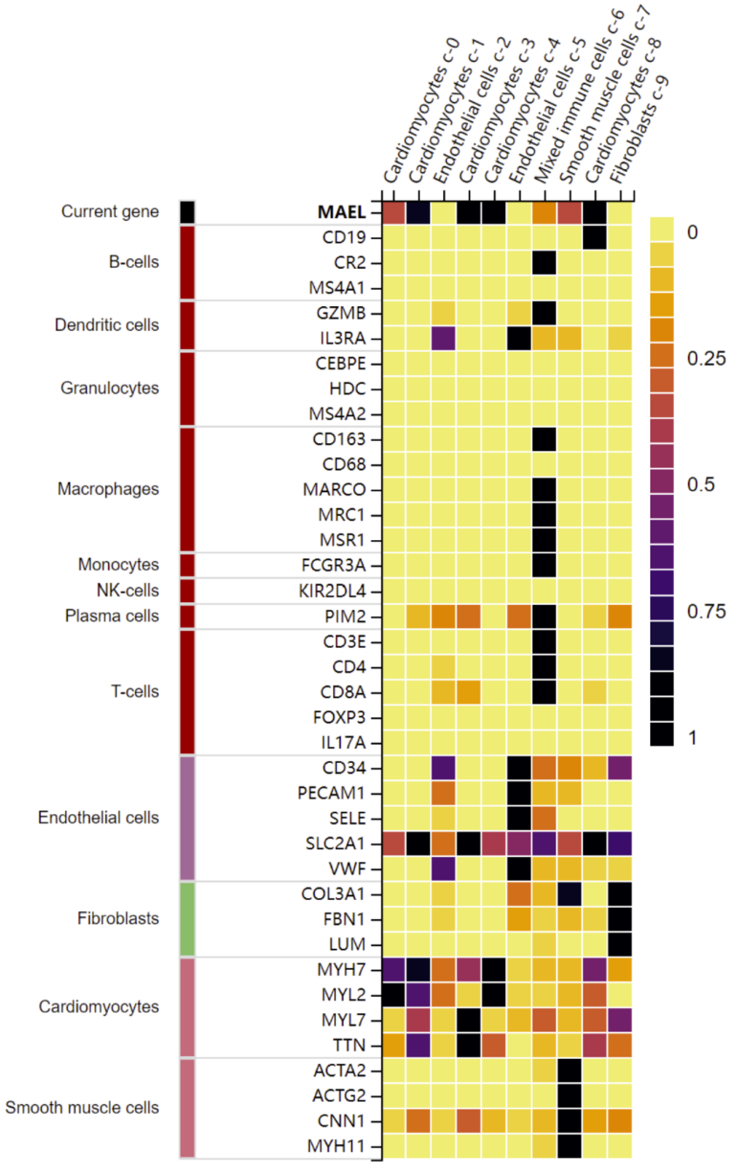

Supplementary Figure 4. mRNA expression of *MAEL* and cell type markers in different single cell type clusters of normal heart muscle (GSE109816). Color legend: fraction of the highest expression.

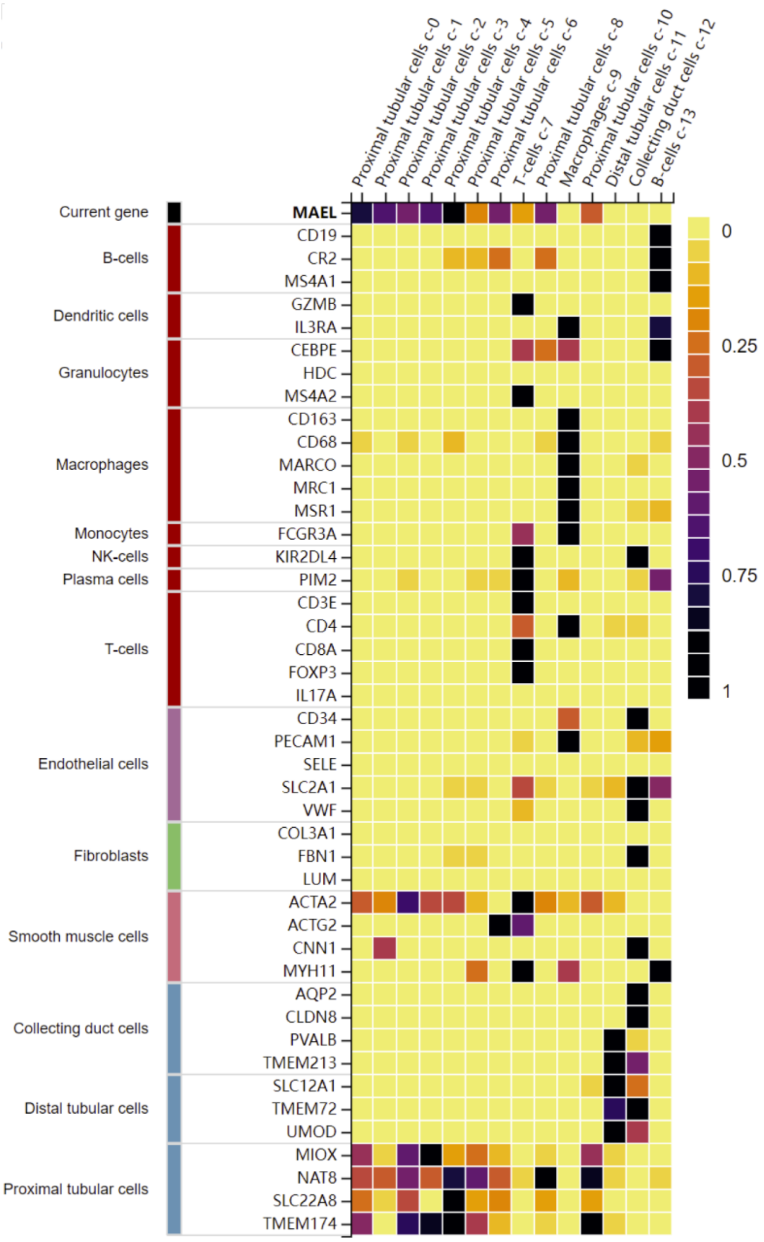

Supplementary Figure 5. mRNA expression of *MAEL* and cell type markers in different single cell type clusters of normal kidney (GSE131685). Color legend: fraction of the highest expression.

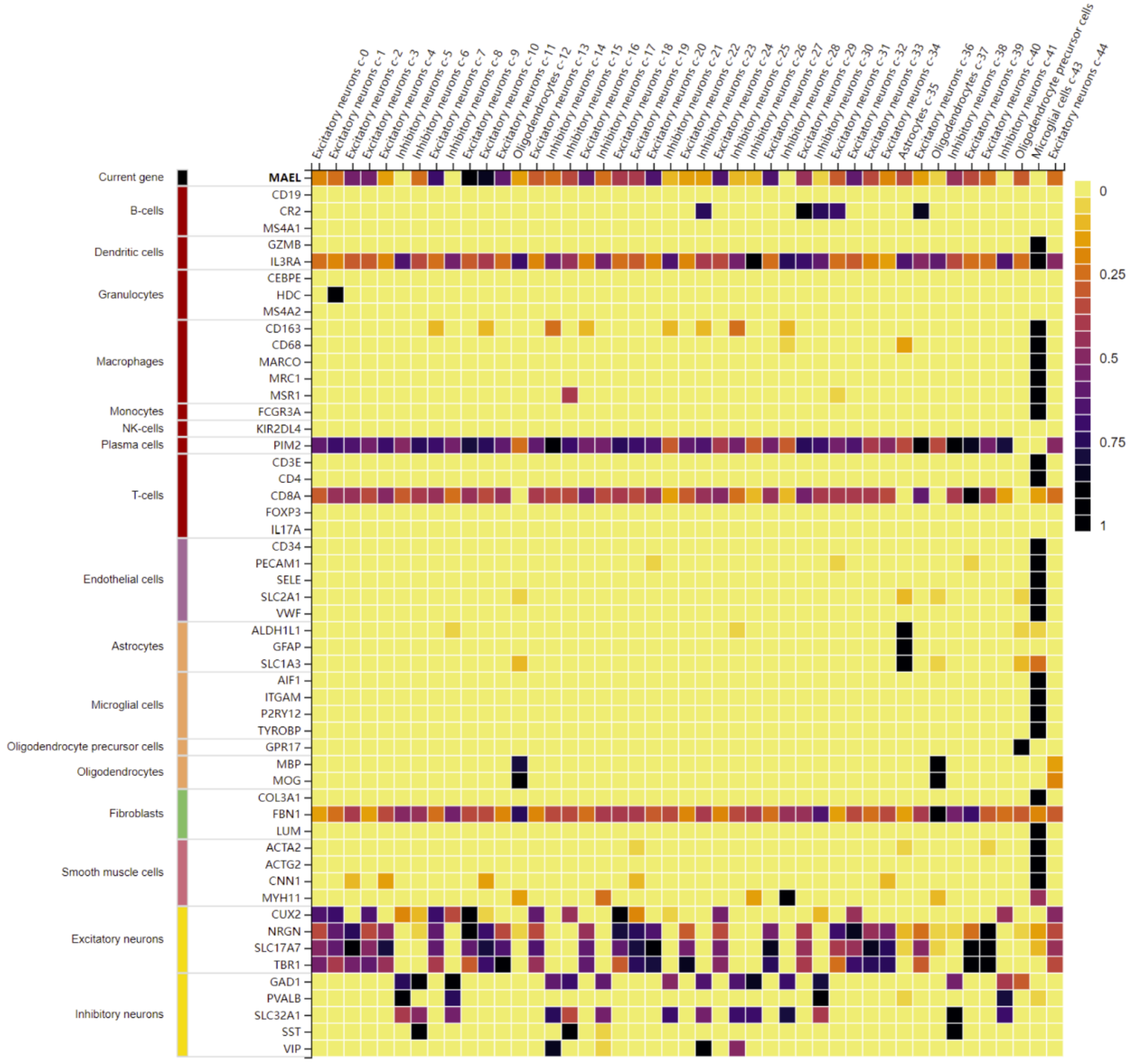

Supplementary Figure 6. mRNA expression of *MAEL* and cell type markers in different single cell type clusters of normal brain (Allen brain map). Color legend: fraction of the highest expression.

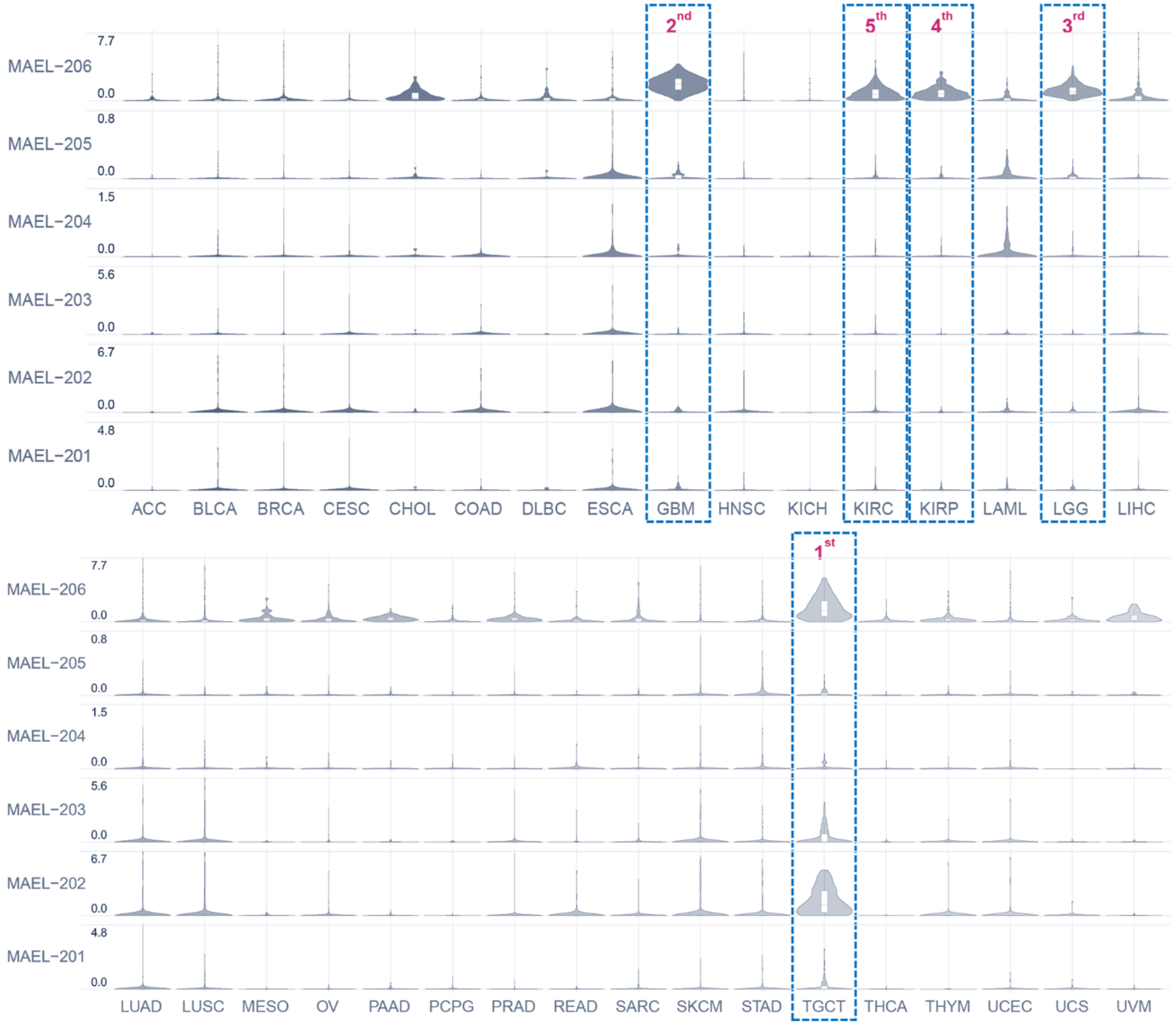

Supplementary Figure 7. mRNA expression of the six transcripts of *MAEL* among 33 types of cancer (unit: log2[TPM+1]).

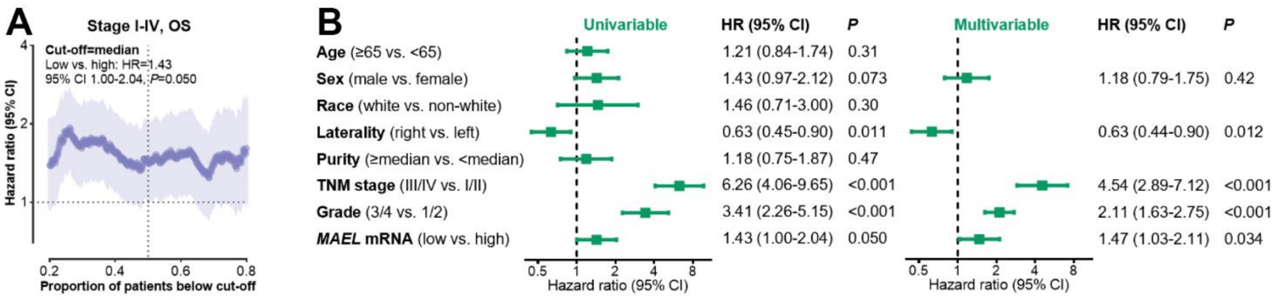

Supplementary Figure 8. Association between *MAEL* expression and recurrence-free survival in the clear cell renal cell carcinomas of the TCGA-KIRC cohort.

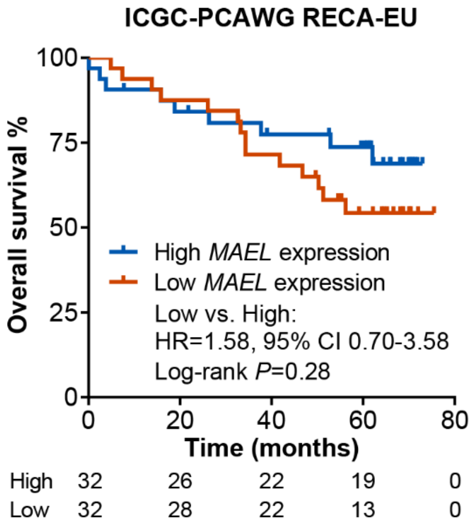

Supplementary Figure 9. Association between *MAEL* expression and overall survival in the clear cell renal cell carcinomas of the ICGC-PCAWG RECA-EU cohort.

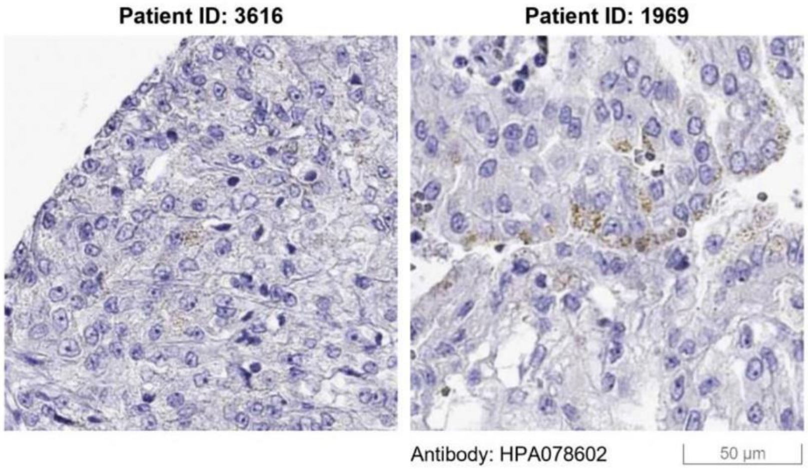

Supplementary Figure 10. Immunohistochemical staining of *MAEL* in ccRCC samples.

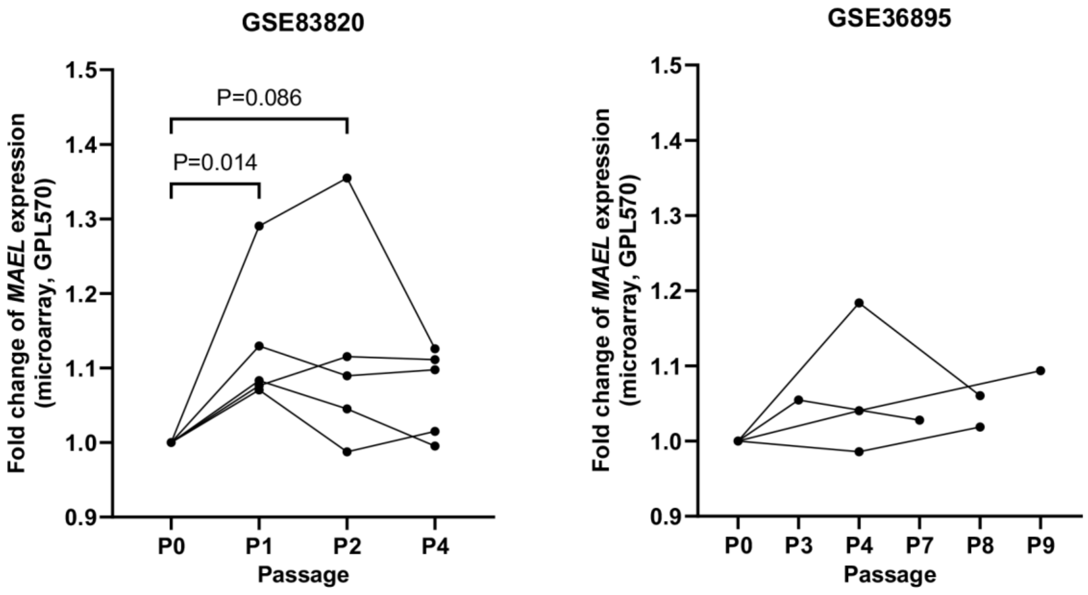

Supplementary Figure 11. Expression of *MAEL* in ccRCC xenograft models.

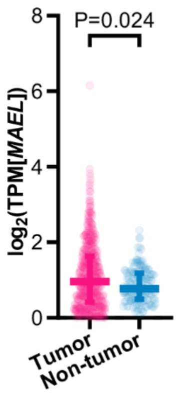

Supplementary Figure 12. *MAEL* expression in clear cell renal cell carcinomas (TCGA-KIRC) and normal kidneys (TCGA-KIRC and GTEx).

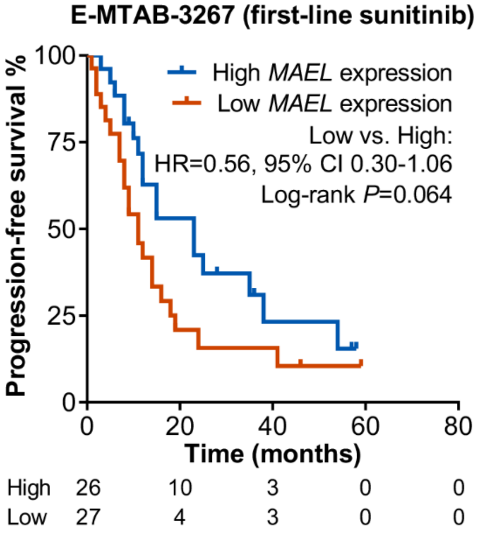

Supplementary Figure 13. High *MAEL* expression was associated with favorable progression-free survival on first-line sunitinib in advanced clear cell renal cell carcinomas (E-MTAB-3267).
