# Supplementary material for: MAEL in human cancers and implications in prognostication and predicting benefit from immunotherapy over VEGFR/mTOR inhibitors in clear cell renal cell carcinoma: a bioinformatic analysis
Source: Aging (Albany NY). 2024 Jan 31;16(3):2090–122. doi: 10.18632/aging.205470 (PMC10911358; doi:10.18632/aging.205470)
Supplement: Supplementary Tables 1 and 2 [file aging-16-205470-s003.pdf]

SUPPLEMENTARY TABLES

Supplementary Table 1. Single cell transcriptomics datasets.

Tissue	Data source	No. of M reads	No. of cells	Correlation with HPA bulk RNA	Reference
Colon	GSE116222	12.9	11167	0.811	Parikh K et al. (2019)
Eye	GSE137537	22.6	20091		Menon M et al. (2019)
Heart muscle	GSE109816	396.7	9182	0.797	Wang L et al. (2020)
Small intestine	GSE125970	59.6	6167		Wang Y et al. (2020)
Kidney	GSE131685	56.2	25279	0.867	Liao J et al. (2020)
Liver	GSE115469	42	8439	0.837	MacParland SA et al. (2018)
Lung	GSE130148	6.9	4599	0.863	Vieira Braga FA et al. (2019)
Placenta	E-MTAB-6701	347	18547	0.879	Vento-Tormo R et al. (2018)
Prostate	GSE117403	177.4	35862	0.756	Henry GH et al. (2018)
Rectum	GSE125970	60.4	3898	0.756	Wang Y et al. (2020)
PBMC	GSE112845	19.4	4972	0.756	Chen J et al. (2018)
Testis	GSE120508	70.5	6490	0.756	Guo J et al. (2018)
Pancreas	GSE131886	92.3	3719	0.829	Qadir MMF et al. (2020)
Skin	GSE130973	56.4	15798	0.756	Solé-Boldo L et al. (2020)
Brain		1357.2	76533	0.661	Allen brain map
Bronchus		85.3	17521		Lukassen S et al. (2020)
Endometrium	GSE111976	624.1	71032	0.807	Wang W et al. (2020)
Skeletal muscle	GSE143704	77.8	22030	0.697	De Micheli AJ et al. (2020)
Ovary	GSE146512	259.2	43636	0.808	Man L et al. (2020)
Adipose tissue	GSE155960	418.5	83536	0.813	Hildreth AD et al. (2021)
Esophagus	159929-GSM4850580	31.5	9117	0.84	He S et al. (2020)
Lymph node	GSE159929-GSM4850583	14.2	7771	0.849	He S et al. (2020)
Bone marrow	GSE159929-GSM4850584	8.6	3230	0.818	He S et al. (2020)
Spleen	GSE159929-GSM4850589	14.8	4512	0.804	He S et al. (2020)
Stomach	GSE159929-GSM4850590	18.9	5318	0.814	He S et al. (2020)
Breast	GSE164898	342.3	47662	0.839	Bhat-Nakshatri P et al. (2021)

Supplementary Table 2. Basic characteristics of the included dataset.

	TCGA-KIRC	ICGC-PCAWG RECA-EU	GDSC	JAVELIN Renal 101	CM-025	E-MTAB-3267	GSE83820	GSE36895
Number of patients/cell lines with mRNA data	522	64	16	726	250	53	5	4
Data used in the present study	Mutation, copy number variation, mRNA, lncRNA, clinicopathological data, OS	mRNA, OS	mRNA, IC50	mRNA, PFS	mRNA, PFS	mRNA, PFS	mRNA	mRNA
Sequencing method of transcriptomic	RNA-seq	RNA-seq	RNA-seq (Illumina HiSeq 2000 or HiSeq 2500)	RNA-seq (Illumina HiSeq 2500)	RNA-seq (Illumina HiSeq 2000 or HiSeq 2500)	Microarray (GPL6244)	Microarray (GPL570)	Microarray (GPL570)
PD-L1 immunohistochemical staining	NA	NA	NA	SP142, immune cell score	NA	NA	NA	NA
Therapy	NA	NA	Targeted agents	Avelumab+Axitinib vs. Sunitinib (1st line)	Nivolumab vs. Everolimus (2nd-3rd line)	Sunitinib (1st line)	NA	NA

Abbreviations: ICGC-PCAWG RECA-EU, International Cancer Genome Consortium-Pan-cancer analysis of whole genomes Renal Cell Carcinoma-Europe; OS, overall survival; PFS, progression-free survival.
