# Supplementary material for: MAEL in human cancers and implications in prognostication and predicting benefit from immunotherapy over VEGFR/mTOR inhibitors in clear cell renal cell carcinoma: a bioinformatic analysis
Source: Aging (Albany NY). 2024 Jan 31;16(3):2090–122. doi: 10.18632/aging.205470 (PMC10911358; doi:10.18632/aging.205470)
Supplement: Supplementary Tables 5-7 [file aging-16-205470-s006.pdf]

**Supplementary Table 5. Abbreviations in the TCGA and the CCLE databases.**

| Abbreviations | Full name | TCGA | CCLE |
|---|---|---|---|
| ACC | Adrenocortical carcinoma | √ | |
| ALL | Acute lymphocytic leukemia | | √ |
| BLCA | Bladder urothelial carcinoma | √ | √ |
| BRCA | Breast invasive carcinoma | √ | √ |
| CESC | Cervical squamous cell carcinoma and endocervical adenocarcinoma | √ | √ |
| CLL | Chronic lymphocytic leukemia | | √ |
| CHOL | Cholangiocarcinoma | √ | |
| COAD | Colon adenocarcinoma | √ | √ |
| DLBC | Diffuse large B cell lymphoma | √ | √ |
| ESCA | Esophageal carcinoma | √ | √ |
| | Ewings sarcoma | | √ |
| GBM | Glioblastoma multiforme | √ | √ |
| HNSC | Head and neck squamous cell carcinoma | √ | √ |
| KICH | Kidney chromophobe | √ | |
| KIRC | Kidney renal clear cell carcinoma | √ | √ |
| KIRP | Kidney renal papillary cell carcinoma | √ | |
| LAML | Acute myeloid leukemia | √ | √ |
| LCML | Chronic myelogenous leukemia | | √ |
| LGG | Brain lower grade glioma | √ | √ |
| LIHC | Liver hepatocellular carcinoma | √ | √ |
| LUAD | Lung adenocarcinoma | √ | √ |
| LUSC | Lung squamous cell carcinoma | √ | √ |
| MB | Medulloblastoma | | √ |
| MESO | Mesothelioma | √ | √ |
| MM | Multiple myeloma | | √ |
| NB | Neuroblastoma | | √ |
| NSCLC | Non-small cell lung cancer | | √ |
| OV | Ovarian serous cystadenocarcinoma | √ | √ |
| PAAD | Pancreatic adenocarcinoma | √ | √ |
| PCPG | Pheochromocytoma and paraganglioma | √ | |
| PRAD | Prostate adenocarcinoma | √ | √ |
| READ | Rectal adenocarcinoma | √ | √ |
| SARC | Sarcoma | √ | √ |
| SCLC | Small cell lung cancer | | √ |
| SKCM | Skin cutaneous melanoma | √ | √ |
| STAD | Stomach adenocarcinoma | √ | √ |
| TGCT | Testicular germ cell tumor | √ | |
| THCA | Thyroid carcinoma | √ | √ |
| THYM | Thymoma | √ | |
| UCEC | Uterine corpus endometrial carcinoma | √ | √ |
| UCS | Uterine carcinosarcoma | √ | |
| UVM | Uveal melanoma | √ | |

**Supplementary Table 6. Features of the six transcripts of *MAEL*.**

| Transcript ID | Name | bp | Protein | Translation ID | Biotype | CCDS | UniProt match | RefSeq match |
|---|---|---|---|---|---|---|---|---|
| ENST00000367870.6 | MAEL-201 | 1831 | 403aa | ENSP00000356844.2 | Protein coding | CCDS65712 | Q96JY0-2 | - |
| ENST00000367872.9 | MAEL-202 | 1737 | 434aa | ENSP00000356846.4 | Protein coding | CCDS1257 | Q96JY0-1 | NM_032858.3 |
| ENST00000447624.1 | MAEL-203 | 1013 | 316aa | ENSP00000402143.1 | Protein coding | | X6RGB1 | - |
| ENST00000487826.1 | MAEL-204 | 416 | No protein | - | Processed transcript | | - | - |
| ENST00000491055.5 | MAEL-205 | 2355 | No protein | - | Processed transcript | | - | - |
| ENST00000622874.4 | MAEL-206 | 1700 | 378aa | ENSP00000482771.1 | Protein coding | CCDS72975 | E9JVC4 | - |

**Supplementary Table 7. Associations of *MAEL* expression with clinicopathological features, copy number variations, and DNA methylation.**

| Variable | KIRC | | TGCT | |
|---|---|---|---|---|
| | P-value | Pearson r | P-value | Pearson r |
| Age | 0.426483266825387 | -0.0324428362771666 | 0.752333563410853 | 0.0270047807184707 |
| Sex | 0.0904257180696398 | NA | NA | NA |
| Race | 0.194246293049914 | NA | 0.872982066854064 | NA |
| Histological grade | 0.021952312664529 | NA | 9.56399423005252E-07 | 0.40542915383908 |
| Pathological T | 0.135787374901966 | NA | 0.177488104232116 | NA |
| Pathologic N | 0.225732314323571 | NA | 0.958850252206529 | NA |
| Pathological M | 0.103451317195837 | NA | NA | NA |
| Tumor stage | 0.0639348682885934 | NA | 0.229241921454448 | NA |
| Copy number variation | 0.0000124053286982694 | 0.190110076688231 | 0.752916482313235 | -0.0259154877503306 |
| cg13081288 | 0.000785685953360897 | -0.180743238710894 | 0.0000628585334375174 | -0.314957369168476 |
| cg08348962 | 0.0492590402535173 | -0.108009393639065 | 0.243763849009391 | -0.0938743944622001 |
| cg18323210 | 0.092512587133264 | -0.0911124474315059 | 0.5440767694976 | 0.0489361999183405 |
| cg03758693 | NA | NA | NA | NA |
| cg11503720 | 0.352805528905452 | 0.0503959163684299 | 0.480610749333206 | 0.0568827237605001 |
| cg15965055 | 0.529796595162321 | 0.034090418549852 | 0.0315425603321009 | 0.172254607829556 |
| cg22603542 | 0.409987244359549 | 0.0446936912631353 | 0.186481189999048 | -0.106325340667379 |
| cg07725889 | NA | NA | NA | NA |
| cg10117884 | 0.897947345270261 | 0.00696064119958166 | 3.94858256423742E-09 | -0.452757600333627 |
| cg18894878 | 0.920711985278922 | -0.00540209688290252 | 6.52822637100908E-16 | -0.610024898231754 |
| cg27053975 | 0.435695401537689 | 0.0422856006668722 | 2.99438405154888E-15 | -0.596408048402836 |
| cg24453820 | 0.301857453783545 | -0.0559901939447633 | 8.67166538012812E-17 | -0.627691428644751 |
| cg23968893 | 0.896436555610462 | -0.00706426328384486 | 1.84345878539201E-15 | -0.60077299206138 |
| cg11336590 | 0.66528813578229 | -0.0236158222038295 | 3.53364947237034E-19 | -0.673975652934597 |
| cg19241352 | 0.0828312211934396 | -0.0939273014761928 | 1.11483104794639E-18 | -0.664515504411586 |
| cg13480774 | 0.709249632452788 | 0.0202341815113491 | 1.49945009591534E-15 | -0.602623339738763 |
| cg24586196 | 0.212520272989992 | -0.0675823337211926 | 2.40573208547595E-16 | -0.618813017897356 |
| cg00001349 | 0.28226677455352 | -0.0583064319125126 | 1.2178606809646E-12 | -0.539768091814994 |
| cg13249256 | 0.283087524604477 | -0.0582072645293623 | 6.21117501997563E-19 | -0.669344119897905 |
| cg17757848 | 0.84317847389987 | -0.0107363993229523 | 2.95924429682114E-19 | -0.675427522082466 |
| cg13212575 | 0.969169533256836 | 0.00209763722753289 | 3.17730546470227E-16 | -0.616374517921076 |
| cg26308818 | 0.596084245542656 | 0.0287606442778004 | 3.39217204532824E-07 | -0.396023176042178 |
